# Mechano-Electrochemical Synergy in Cellulose@MOF Scaffold-Based Asymmetric Electrolyte for Stable Solid-State Lithium Metal Batteries

**DOI:** 10.1007/s40820-025-02039-x

**Published:** 2026-01-15

**Authors:** Wanqing Fan, Xuetao Shi, Ying Huang, Kaihang She, Bowei Song, Zheng Zhang

**Affiliations:** 1 The MOE Key Laboratory of Material Physics and Chemistry Under Extraordinary Conditions, Ministry of Education, School of Chemistry and Chemical Engineering, Northwestern Polytechnical University, Xian, 710072 People’s Republic of China; 2 State Key Laboratory of Efficient Production of Forest Resources, Beijing Key Laboratory of Lignocellulosic Chemistry, Beijing Forestry University, Beijing, 100083 People’s Republic of China

**Keywords:** Solid-state lithium metal batteries, Asymmetric composite solid-state electrolyte, Elastic modulus, Pouch cells

## Abstract

The application of polymer electrolytes is expected to revitalize solid-state lithium metal batteries (SSLMBs) with high energy density and enhanced safety. However, practical deployment faces challenges from inadequate mechanical properties of electrolyte and unstable interfaces in high-voltage SSLMBs. Herein, we design an asymmetric composite solid-state electrolyte (ACSE) composed of a cellulose framework in situ self-assembled with zeolitic imidazolate framework nanosheets (CP@MOF) embedded in a polymer matrix. The CP@MOF network provides the electrolyte with an elastic modulus of 1.19 GPa, effectively resisting Li dendrite penetration. Furthermore, theoretical calculations guided the compositional design of ACSE to address asynchronous interfacial requirements at cathode/electrolyte and anode/electrolyte interfaces, facilitating stable interphase formation and thus ensuring prolonged cycling of SSLMBs. Consequently, Li symmetric cells achieve extended cycling stability (> 5000 h) with minimal polarization. The NCM811|Li full cell maintains 84.9% capacity retention after 350 cycles. Notably, assembled NCM811 pouch cells deliver practical energy densities of 337.9 Wh kg^−1^ and 711.7 Wh L^−1^, demonstrating exceptional application potential. This work provides novel insights into the application of ACSEs for high-energy–density SSLMBs.

**Supplementary Information:**

The online version contains supplementary material available at 10.1007/s40820-025-02039-x.

## Highlights

- A structurally simple asymmetric solid-state electrolyte successfully stabilizes the interface between lithium metal and high-voltage cathodes in solid-state lithium metal batteries.
- Environmentally friendly cellulose provides high mechanical support, while layered self-assembled metal–organic frameworks restrict TFSI⁻, efficiently promoting Li⁺ transport.
- The assembled pouch cell exhibited a high gravimetric/volume energy density of 337.9 Wh kg^−1^/711.7 Wh L^−1^.

**Supplementary Information:**

The online version contains supplementary material available at 10.1007/s40820-025-02039-x.

## Graphical abstract

**Figure:**
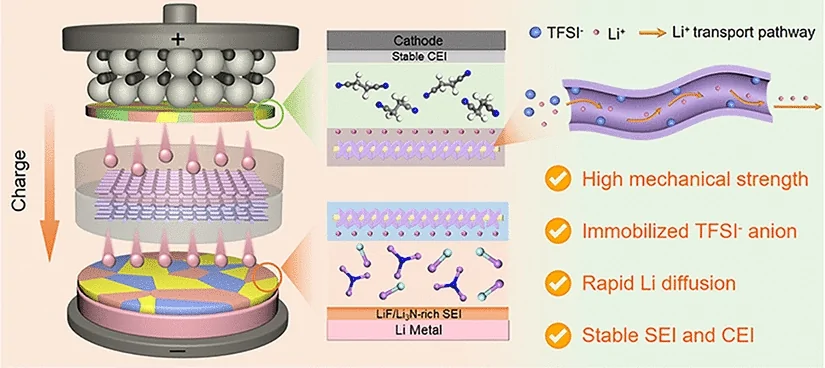

## Introduction

Solid-state lithium metal batteries (SSLMBs), as a pivotal direction for next-generation high-performance battery technologies, significantly enhance energy density by employing lithium (Li) metal anodes with high theoretical specific capacity (3860 mAh g^−1^), while replacing flammable liquid electrolytes with solid-state electrolytes (SSEs) fundamentally improves battery safety [1, 2]. The performance of SSLMBs strongly depends on the comprehensive properties of SSEs. Ideal SSEs should simultaneously possess excellent mechanical properties (to suppress Li dendrite penetration) and high ionic conductivity (to facilitate efficient ion transport) [3–5]. Among various SSEs, poly(ethylene oxide) (PEO)-based electrolytes have been extensively studied due to their flexibility, ease of processing, and good electrode interfacial compatibility [6]. However, their low room-temperature ionic conductivity (∼10^–6^–10^–7^ S cm^−1^), low Li^+^ transference number, and insufficient mechanical strength severely limit practical application [7]. To overcome these limitations, strategies such as developing cross-linked polymer networks [8, 9], single-ion conducting polymers [10, 11], and incorporating inorganic fillers [12, 13] have been explored. These approaches enhance ionic conductivity and electrochemical stability while maintaining the flexibility of the matrix. Furthermore, three-dimensional (3D) structures such as poly(p-phenylene benzobisoxazole) (PBO) [14] nanofibers and aramid nanofiber (ANF) aerogels [15] have been employed as mechanical reinforcement frameworks to improve the mechanical properties of composite solid electrolytes (CSEs). However, the introduction of these non-lithium-conducting structures often sacrifices ionic conductivity, leading to a trade-off between ion transport and mechanical strength [16]. Meanwhile, such inert host materials cannot alleviate ion concentration polarization. Therefore, the coordinated optimization of ionic conductivity and mechanical properties, along with rational design of CSEs, remains a major challenge. It must be emphasized that, while continuing to pursue excellent electrochemical and mechanical performance, equal attention must be paid to practical constraints such as cost control, environmental compatibility, and scalable manufacturing, which are essential pathways toward practical application [17–19].

Cellulose is the most abundant natural polymer on Earth, offering advantages such as wide availability, low cost, excellent mechanical properties, and good thermal stability. The abundant polar functional groups (e.g., −OH and −O−) on its molecular chains facilitate ion transport [20], while its high-strength fibrous network can serve as a stable 3D framework material, effectively enhancing the mechanical strength of SSEs and suppressing Li dendrite penetration. Furthermore, cellulose can also be used as a directional template to guide the controlled growth of functional materials [21]. In recent years, metal–organic frameworks (MOFs) have demonstrated great potential in promoting Li^+^ migration and guiding uniform Li deposition due to their high porosity, large specific surface area, and tunable pore structures [22]. Therefore, the in situ incorporation of MOF nanostructures into cellulose frameworks to construct multi-functional composite hosts is considered a feasible strategy to address current challenges in polymer electrolytes.

Another critical bottleneck hindering the practical application of SSLMBs lies in the interfacial issues between the electrolyte and electrodes. SSLMBs feature two critical internal interfaces: the electrolyte|Li metal interface (anode side) and the cathode|electrolyte interface (cathode side). On the Li anode side, uncontrolled growth of Li dendrites may occur, potentially leading to battery short circuits and even safety incidents [23–25]. On the cathode side, the electrolyte must possess a wide electrochemical window to match high-voltage cathode materials [26, 27]. However, a single electrolyte system simultaneously satisfying all the aforementioned performance requirements remains challenging to achieve. Therefore, designing ACSEs with specialized compositional structures to synergistically address interfacial issues on both anode and cathode sides is crucial [28]. For example, Yao et al. [29] designed an ACSE comprising a high-voltage stable layer and a Li anode compatible layer, achieving high ionic conductivity and excellent electrochemical stability through structural design. Lv et al. [30] introduced distinct modification layers at the anode and cathode interfaces, effectively reducing the reactivity at the anode interface and enhancing the high-voltage tolerance at the cathode interface, thereby enabling stable cycling of high-voltage SSLMBs. Despite significant progress in the aforementioned studies, ACSEs fabricated via layer-by-layer stacking exhibit inherent limitations. Not only potentially introduce additional interfacial contact resistance between layers, but also inevitably increase the overall electrolyte thickness. Moreover, such electrolytes often suffer from insufficient mechanical strength and lack robust supporting structures, making it difficult to construct effective mechanical barriers to suppress Li dendrite penetration. Consequently, this restricts their ability to maintain long-term stable Li plating/stripping cycling.

**Fig. 1:**
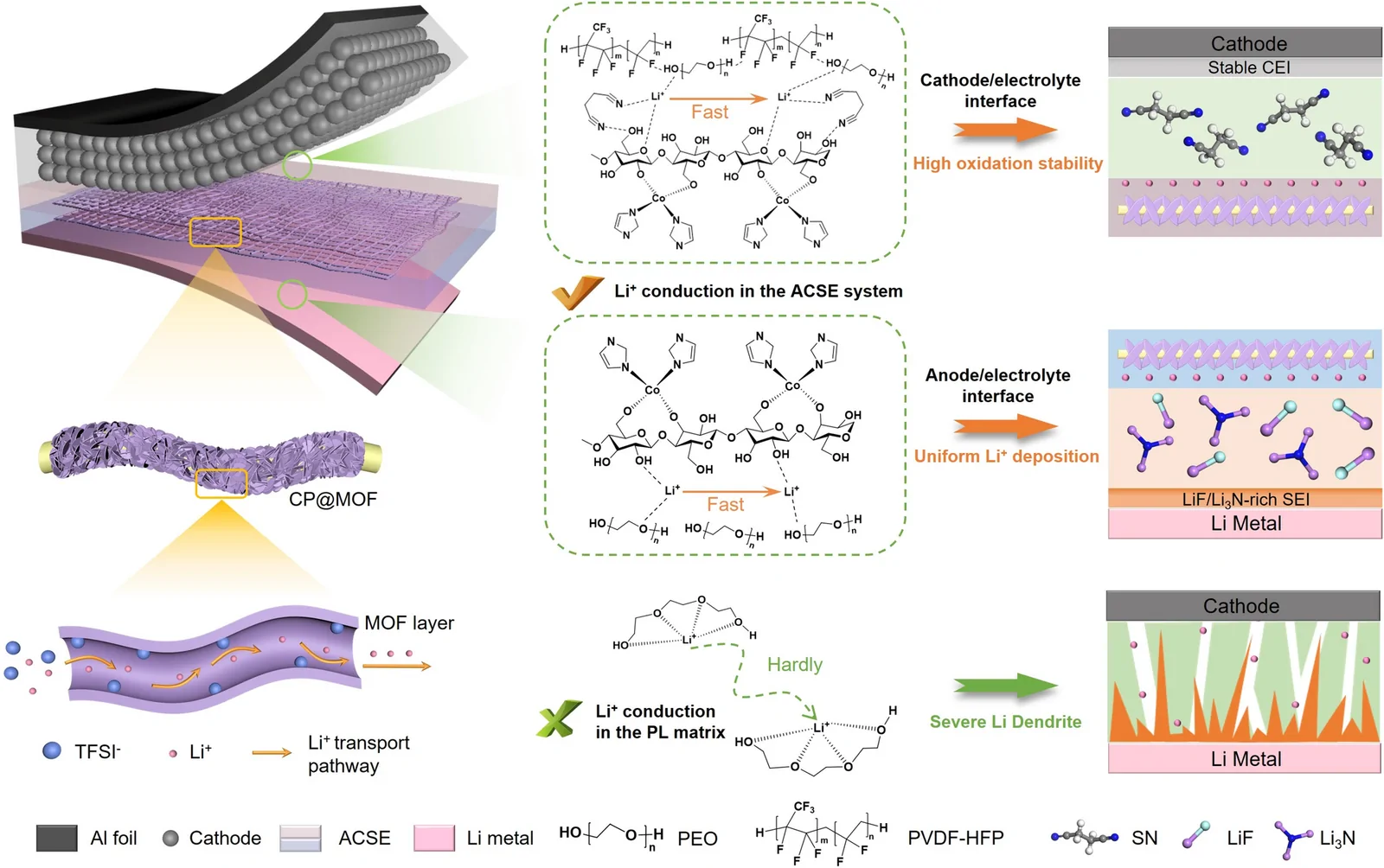
Schematic structure of ACSE electrolyte and Li^+^ conduction process compared with PL matrix

Herein, we design an ACSE based on an environmentally friendly cellulose paper (CP) 3D rigid skeleton (Fig. 1). A hierarchically self-assembled CP@MOF composite network is constructed by in situ growing 2D MOF nanosheets along the fiber surfaces. Functionalized electrolyte layers are built on both sides: the anode side employs a PEO matrix containing ionic liquids (ILs) to form a PLCM/IL composite layer, while the cathode side introduces a modified layer composed of PEO, poly(vinylidene fluoride-co-hexafluoropropylene) (PVDF-HFP), and succinonitrile (SN). Benefiting from the mechanical support of the CP skeleton, the ACSE combines an ultrathin thickness (~ 32.5 μm) with excellent mechanical properties (elastic modulus of 1.19 GPa). Furthermore, the asymmetric structure design enables partitioned optimization for the anode and cathode. On the anode side, densely arranged MOF nanosheets effectively block TFSI^−^ anion migration and suppress space charge layer formation. On the cathode side, PVDF-HFP and SN synergistically enhance interfacial compatibility. Theoretical calculations and experimental characterizations demonstrate that the prepared ACSE exhibits high ionic conductivity (4.39 × 10^–4^ S cm^−1^), significant Li dendrite suppression capability, and stable solid electrolyte interphase (SEI)/ cathode electrolyte interface (CEI) interfaces. The assembled NCM811|ACSE|Li full cell achieves an 84.9% capacity retention after 350 cycles. Moreover, the NCM811|Li pouch cell equipped with this ACSE exhibits high energy density (337.9 Wh kg^−1^/711.7 Wh L^−1^) and maintains output even under extreme conditions such as bending and piercing, highlighting its engineering application potential. These findings provide new insights for interface optimization in high-voltage SSLMBs.

## Experimental Section

The preparation process of CP@MOF typically involves dissolving 0.58 g of Co(NO_3_)_2_·6H_2_O in 40 mL of water to prepare Solution A, and separately dissolving 1.31 g of 2-MIm in 40 mL of water to prepare Solution B. After stirring each solution for 20 min, Solution B is quickly poured into Solution A and stirred for an additional 5 min. The mixture is then allowed to stand for 2 h to allow CP to settle at the bottom. The other side of the cellulose paper is subjected to ultrasonic cleaning with ethanol, followed by a secondary deposition step by repeating the above process. The in situ synthesized CP@MOF film is subsequently cleaned ultrasonically with ethanol and vacuum-dried at 60 °C for 12 h for further use. PEO and LiTFSI (EO:Li = 15:1) were dissolved in acetonitrile, and 10 wt% ILs was added with continuous stirring for 12 h and then poured into PTFE molds and then into CP@MOF films. Another PVDF-HFP was dissolved in DMF, PEO, and LiTFSI with LiDFOB (the mass of LiTFSI remains unchanged, with a mass ratio of LiODFB to LiTFSI at 1:2) dissolved in acetonitrile (1:4 mass ratio of PVDF-HFP: PEO), followed by the addition of 10 wt% of SN, all mixed for 12 h with continuous stirring and then poured onto the other side of the CP@MOF film. The film was dried in a vacuum-drying dish for 24 h and then transferred to a vacuum oven at 60 °C for an additional 24 h of drying, ultimately yielding the ACSE film.

## Results and Discussion

### Morphological and Physicochemical Characteristics

**Fig. 2:**
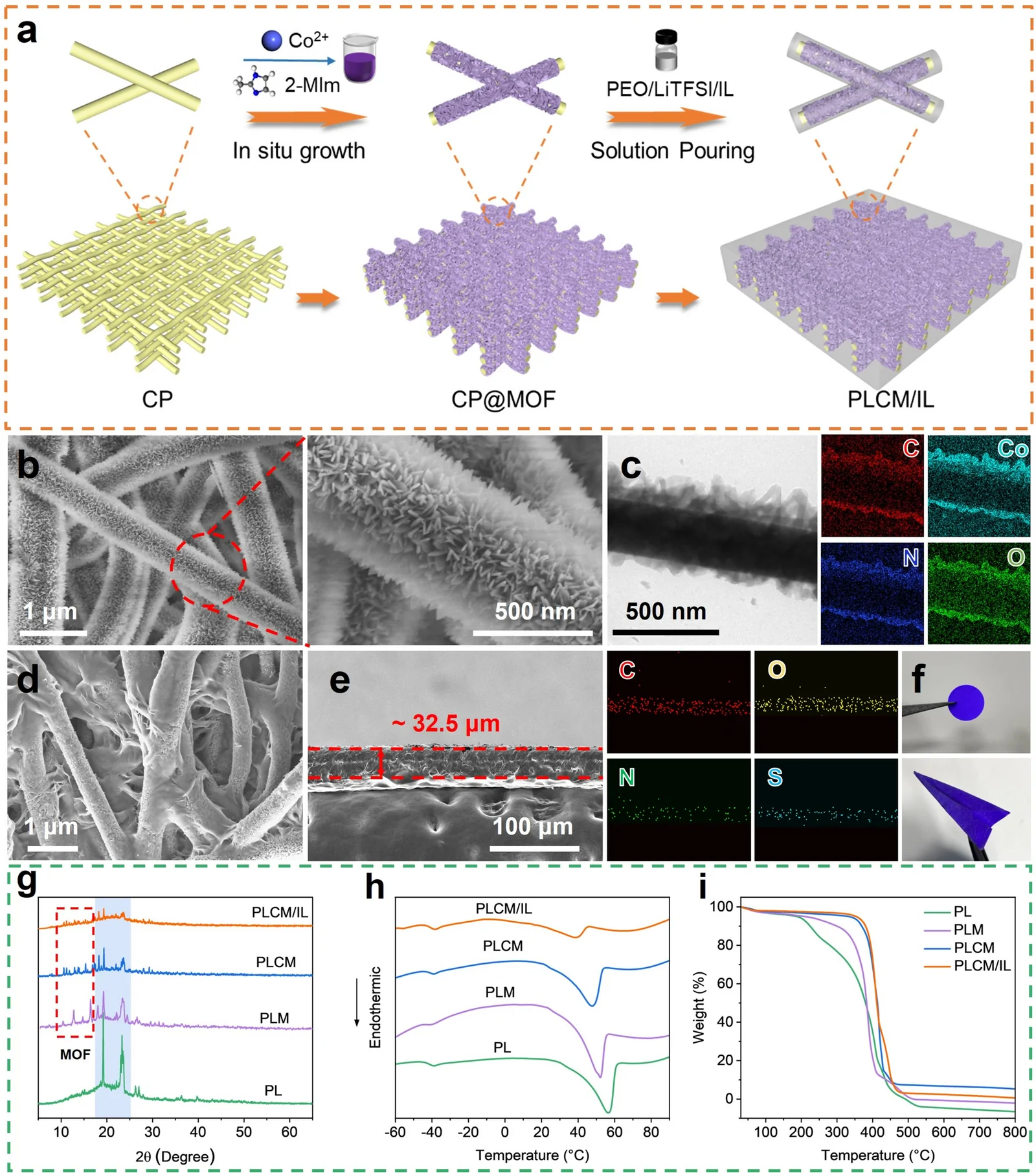
**a** Schematic diagram of PLCM/IL electrolyte preparation process. **b** SEM images, **c** TEM image and EDS mapping images of CP@MOF fiber. SEM images of the **d** surface and **e** cross section of the PLCM/IL and EDS mapping of the cross section of PLCM/IL. **f** Optical photographs of the PLCM/IL. **g** XRD patterns, **h** DSC curves and **i** TGA curves of PL, PLM, PLCM and PLCM/IL

The design and preparation process of the PLCM/IL composite solid electrolyte based on self-assembled MOF networks are illustrated in Fig. 2a. Environmentally friendly cellulose paper (CP) is selected as the 3D scaffold material to enhance the mechanical properties of the electrolyte. This scaffold consists of an interconnected fiber network with a thickness of 31.2 μm (Fig. S1). The micro-sized porous structure formed between the fibers provides an ideal framework for the sufficient infiltration of the subsequent polymer matrix. MOF (ZIF-67) nanosheets were successfully constructed on the surface of CP through an in situ growth method, leading to the creation of a layered self-assembled CP@MOF composite structure. At the macroscopic scale, SEM images confirm the uniform epitaxial growth of MOF nanosheets on the surfaces of CP fibers, resulting in a complete two-dimensional coating structure (Fig. 2b). Notably, the MOF nanosheets are orderly arranged along the fiber axis at the micro–nanoscale, establishing continuous ion transport channels. Importantly, the nanopores of MOF can selectively hinder the migration of large-sized anionic clusters through spatial confinement effects, thereby enhancing the mobility of Li^+^ [31]. The MOF nanosheets recovered from the growth solution exhibit a typical two-dimensional leaflike morphology (Fig. S2), with a Brunauer–Emmett–Teller (BET) surface area of 1189.3 m^2^ g^−1^ (Fig. S3); this high specific surface area provides ample contact area for subsequent interfacial interactions. TEM analysis further confirms the continuity of the self-assembled CP@MOF structure (Fig. 2c), which is crucial for constructing a long-range ion conduction network. EDS mapping results validate the complete coating of CP fibers by MOF nanosheets and their elemental distribution characteristics, while X-ray diffraction (XRD) patterns confirm the successful crystallization of the MOF (Fig. S4). The ionic liquid (IL)-doped polymer matrix was infused into the CP@MOF scaffold via a solution impregnation method, ultimately yielding the PLCM/IL composite solid electrolyte. SEM surface morphology shows that the polymer matrix completely fills the inter-fiber pores, forming a continuous phase structure (Fig. 2d). Cross-sectional analysis indicates that the overall density of the electrolyte is significantly improved, with a thickness controlled at 32.5 μm (Fig. 2e). Elemental mapping reveals a uniform distribution of C, O, N, and S elements, confirming the good compatibility of the components. Notably, the PLCM/IL electrolyte exhibits excellent foldability (capable of being folded into a paper airplane shape, Fig. 2f), showcasing outstanding mechanical stability and flexibility features.

The phase composition evolution of the CSEs was investigated using XRD analysis (Fig. 2g). The pure polyethylene oxide (PEO)-based electrolyte (PL) exhibits typical crystalline diffraction peaks at 19.2° and 23.4°. Notably, upon the introduction of MOF nanoscale fillers and the CP@MOF 3D network, the intensity of the characteristic peaks of PEO in both PLCM and PLCM/IL systems significantly decreases. This considerable increase in amorphization is beneficial for enhancing ionic conductivity [32]. The aforementioned changes in crystallinity were further validated by differential scanning calorimetry (DSC) analysis (Fig. 2h). The glass transition temperatures (T_g_) of PLCM and PLCM/IL are both lower than that of the pure PL electrolyte. Importantly, the XRD patterns clearly show the characteristic diffraction peaks of MOF, confirming the retention of the MOF framework structure during the composite process. To further evaluate the thermal reliability of the CSEs, thermogravimetric analysis (TGA) was employed for quantitative characterization of thermal stability (Fig. 2i). Under a nitrogen atmosphere, the initial decomposition temperatures (T_d_) of PLCM and PLCM/IL reach 322 and 330 °C, respectively, significantly higher than that of pure PL (191 °C) and PLM (257 °C) (Table S1). Furthermore, high-temperature dimensional stability tests (Fig. S5) indicate that PL and PLM undergo significant softening or even liquefaction in the temperature range of 90–120 °C, while PLCM/IL maintains structural integrity at 150 °C, demonstrating excellent high-temperature mechanical stability. This characteristic is of great significance for the thermal safety protection of SSLMBs.

### Mechanical and Electrochemical Characterizations

**Fig. 3:**
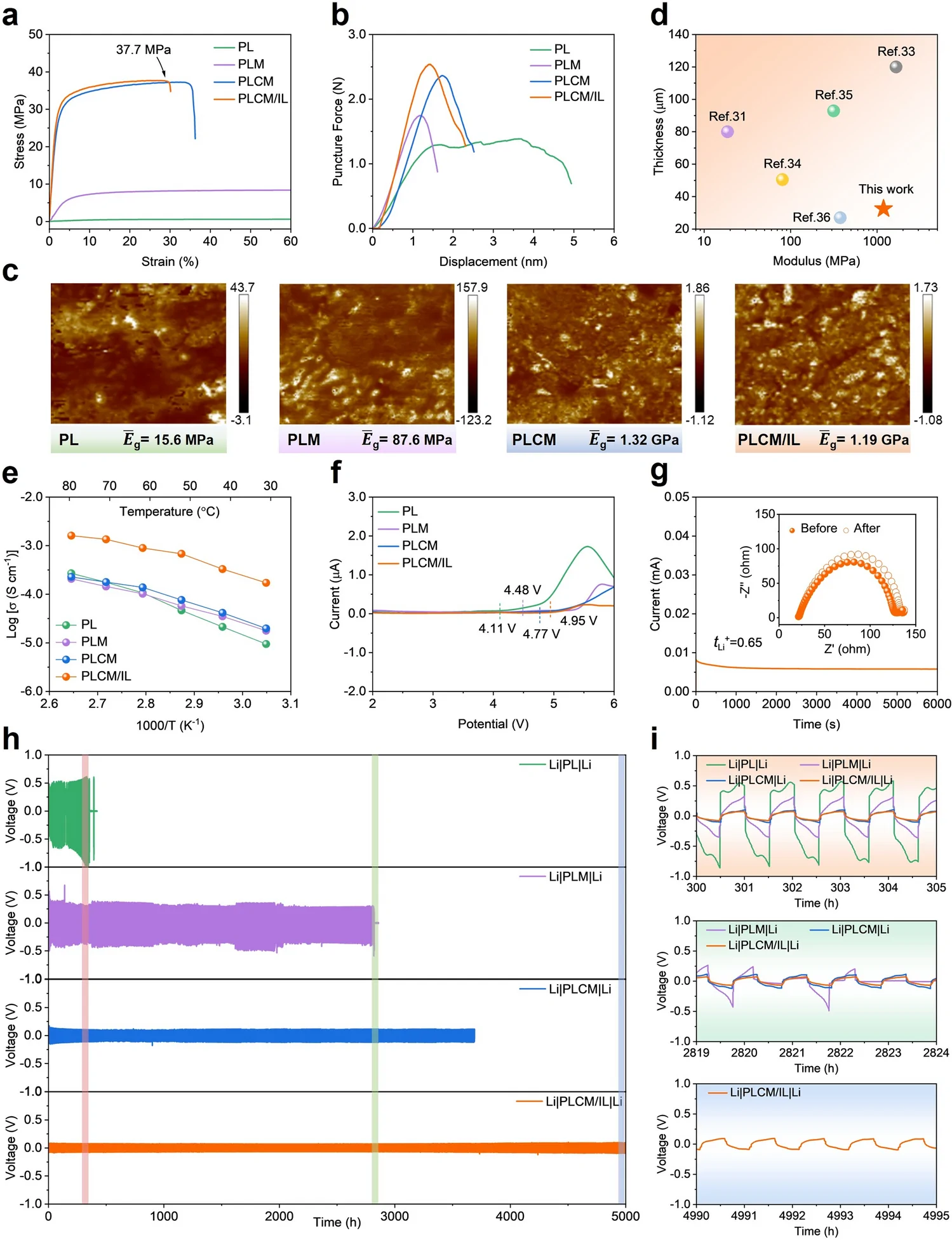
**a** Stress–strain curves, **b** puncture strength of PL, PLM, PLCM and PLCM/IL. **c** Elastic modulus mapping of CSEs obtained by AFM. **d** Comparison of the Young's modulus of recently reported CSEs with the thickness of the electrolyte. **e** Ion conductivity and **f** linear sweep voltammetry curves of PL, PLM, PLCM and PLCM/IL. **g** Li^+^ transfer number of PLCM/IL. **h** Long-term cycling performance of Li|Li symmetrical cells at a current density of 0.2 mA cm^−2^. **i** Local magnification curves of Li|Li symmetric cells at different positions

Based on the practical application requirements of CSEs in SSLMBs, a systematic assessment was conducted on the mechanical reliability of the electrolyte and its ability to suppress dendrite growth. Multi-scale mechanical characterization revealed that the CP@MOF 3D network significantly enhances the mechanical properties of the polymer matrix. Macroscopic mechanical tests show that the tensile strengths of PLCM and PLCM/IL reach 37.7 and 32.2 MPa, respectively, which are markedly higher than those of the PL and PLM electrolytes (Fig. 3a). Additionally, the Young's modulus (Fig. S6) calculated from the tensile curves further demonstrates that the modulus of PLCM/IL (1.26 GPa) is approximately 108 times higher than that of PL (11.6 MPa), confirming the reinforcing effect of the 3D continuous framework on the stiffness of the matrix. Additionally, dynamic mechanical analysis (DMA) was employed to investigate the variation of electrolyte modulus with temperature. As shown in Fig. S7, the storage modulus of PL and PLM drops sharply between 65 and 75 °C. This can be ascribed to the structural softening of the PL matrix upon reaching its transition temperature. In contrast, after incorporating the CP@MOF supporting structure, the modulus attenuation of the PLCM and PLCM/IL systems is significantly mitigated. Even when the temperature rises to 100 °C, their storage moduli remain at relatively high levels of 264 and 92 MPa, respectively, demonstrating excellent thermomechanical stability.

The puncture strength test quantifies the electrolyte's resistance to Li dendrite penetration (Fig. 3b), with PLCM and PLCM/IL providing puncture strengths of 2.53 N and 2.36 N, respectively, exceeding those of PL (1.74 N) and PLM (1.28 N). To elucidate the micro-mechanical enhancement mechanism, atomic force microscopy (AFM) combined with the Derjaguin–Muller–Toporov (DMT) model was employed to quantitatively analyze the modulus distribution of the multi-phase system (Figs. 3c and S8). The elastic modulus (E_g_) of the pure polymer phase (PL) is only 15.6 MPa, while the incorporation of MOF particles raises the E_g_ of PLM to 87.6 MPa. Notably, the 3D CP@MOF network allows PLCM/IL to attain an E_g_ of 1.19 GPa, which is highly consistent with the values from macroscopic modulus testing. This confirms the synergistic matching of mechanical properties from the nanoscale to the macroscopic scale. This multi-level enhancement effect can be attributed to: 1) the strong interfacial coupling between MOF nanosheets and polymer chains, which restricts chain slippage; and 2) the effective load transfer enabled by the CP fiber network structure. Importantly, PLCM/IL achieves a GPa-level modulus while maintaining an ultrathin thickness of 32.5 μm, significantly outperforming reported analogous ultrathin CSEs systems (Fig. 3d, Table S2) [31, 33–36]. These outstanding mechanical properties provide a critical material foundation for constructing ultrathin SSLMBs with high energy density and safety.

Electrochemical characterization revealed the various electrochemical properties of the CSEs, focusing on key metrics such as ionic conductivity (IC), Li^+^ transference number (*t*_Li_^+^), electrochemical stability window (ESW), and Li plating/stripping kinetics. Temperature-dependent ionic conductivity analysis and Nyquist plots indicated significant differences in ionic conduction behaviors across the different systems (Figs. 3e and S9). The ionic conductivity of PL at 30 °C is only 9.45 × 10^–6^ S cm^−1^, which is closely related to the restricted segmental movement due to its high crystallinity. After the introduction of discrete MOF particles, the IC of PLM increased to 1.78 × 10^–5^ S cm^−1^. However, the local aggregation effects of the MOF particles hindered the formation of long-range ordered ionic transport pathways. With the structural design of the layered self-assembled CP@MOF 3D network, the IC of PLCM further increased to 1.97 × 10^–5^ S cm^−1^, benefiting from three synergistic mechanisms: 1) the spatial confinement effect of the MOF nanopores suppresses the recrystallization of PEO chains; 2) the MOF–polymer interface creates a rapid Li^+^ transport pathway; and 3) the 3D interpenetrating network increases the effective transport interfacial area. Upon further incorporation of ILs to form PLCM/IL, the IC surged to 1.72 × 10^–4^ S cm^−1^, representing nearly a two-order of magnitude enhancement compared to the PL system, attributable to the plasticizing effect of Ils [37, 38].

Linear sweep voltammetry (LSV) tests demonstrated that the ESW of PLCM/IL expanded to 4.95 V (vs. Li^+^/Li), a significant increase compared to PL (4.11 V), indicating that the CP@MOF network can enhance the electrochemical stability of the electrolyte (Fig. 3f). This wide voltage window characteristic enables compatibility with high-voltage cathode materials, laying the foundation for constructing high-energy–density SSLMBs. Polarization–impedance testing quantitatively analyzed the *t*_Li_^+^ (Fig. 3g). The calculated transference number of PLCM/IL is 0.65, significantly higher than that of PL (0.27) and PLM (0.42) (Fig. S10), and this value notably exceeds recent reports (Fig. S11) [31, 33, 39–45]. This result demonstrates that the formation of a 3D continuous space by the CP@MOF, in conjunction with the constrained TFSI^−^ anions, provides a continuous and rapid pathway for Li^+^ transport [23].

A mechanical–electrochemical synergistic regulation strategy based on CSEs was systematically investigated to reveal the suppression mechanisms of Li dendrite growth in the PLCM/IL system using various characterization techniques. The long cycle stability of symmetric batteries (Li|CSEs|Li) was evaluated using a constant current polarization method. As shown in Fig. 3h, the symmetric Li battery equipped with the PLCM/IL electrolyte achieved over 5000 h of stable cycling at a current density of 0.2 mA cm^−2^, with the polarization voltage remaining below 150 mV (Fig. S12) and no short-circuit failures occurring. In contrast, the other three electrolytes exhibited poor cycling stability and higher overpotentials. Specifically, the Li|PL|Li battery experienced micro-short-circuiting after 140 h of cycling and was completely short-circuited by 350 h. After the introduction of MOF particles, the Li|PLM|Li battery short-circuited after approximately 2820 h (Fig. 3i), accompanied by significant voltage fluctuations and increased overpotential. Although the PLCM electrolyte reduced the overpotential of the Li symmetric battery, the battery failed after 3700 h of cycling. Clearly, compared to electrolytes without the CP@MOF network, both the PLCM and PLCM/IL electrolytes demonstrated superior cycling stability and smaller polarization potentials. Additionally, the rate performance of the PLCM/IL symmetric Li battery was further tested (Fig. S13). As the current density increased, the voltage profiles exhibited a steady upward trend, and upon restoring the current density to a lower value, the PLCM/IL electrolyte showed similar overpotential values compared to previous measurements (Fig. S14), ensuring consistency during the Li plating/stripping process. To further validate the ability of the electrolyte to suppress Li dendrite formation, SEM characterization was conducted on Li anodes after cycling for 300 and 1000 h with the four electrolytes (Fig. S15). The results indicated that the Li anode surface in the PLCM/IL battery was smooth and exhibited no significant dendrite formation, while the surfaces of the Li metal electrodes with PL, PLM, and PLCM electrolytes showed varying degrees of loose Li dendrites, with PLCM featuring relatively fewer dendrites. Moreover, by comparing the electrochemical impedance spectra (EIS) of Li stripping cycles before and after cycling (Fig. S16), the Li symmetric battery with the PLCM/IL electrolyte displayed lower initial impedance and a smaller trend in impedance variation. These results confirm that the high modulus and high mechanical strength of the PLCM and PLCM/IL electrolytes can effectively suppress the growth of Li dendrites. Furthermore, the incorporation of ILs significantly enhanced the ionic conductivity of the electrolyte, facilitating rapid Li^+^ transport and markedly improving the stability of the electrode/electrolyte interface, thereby achieving a longer cycling lifespan.

### Interface Analysis and Li^+^ Transport Simulation

**Fig. 4:**
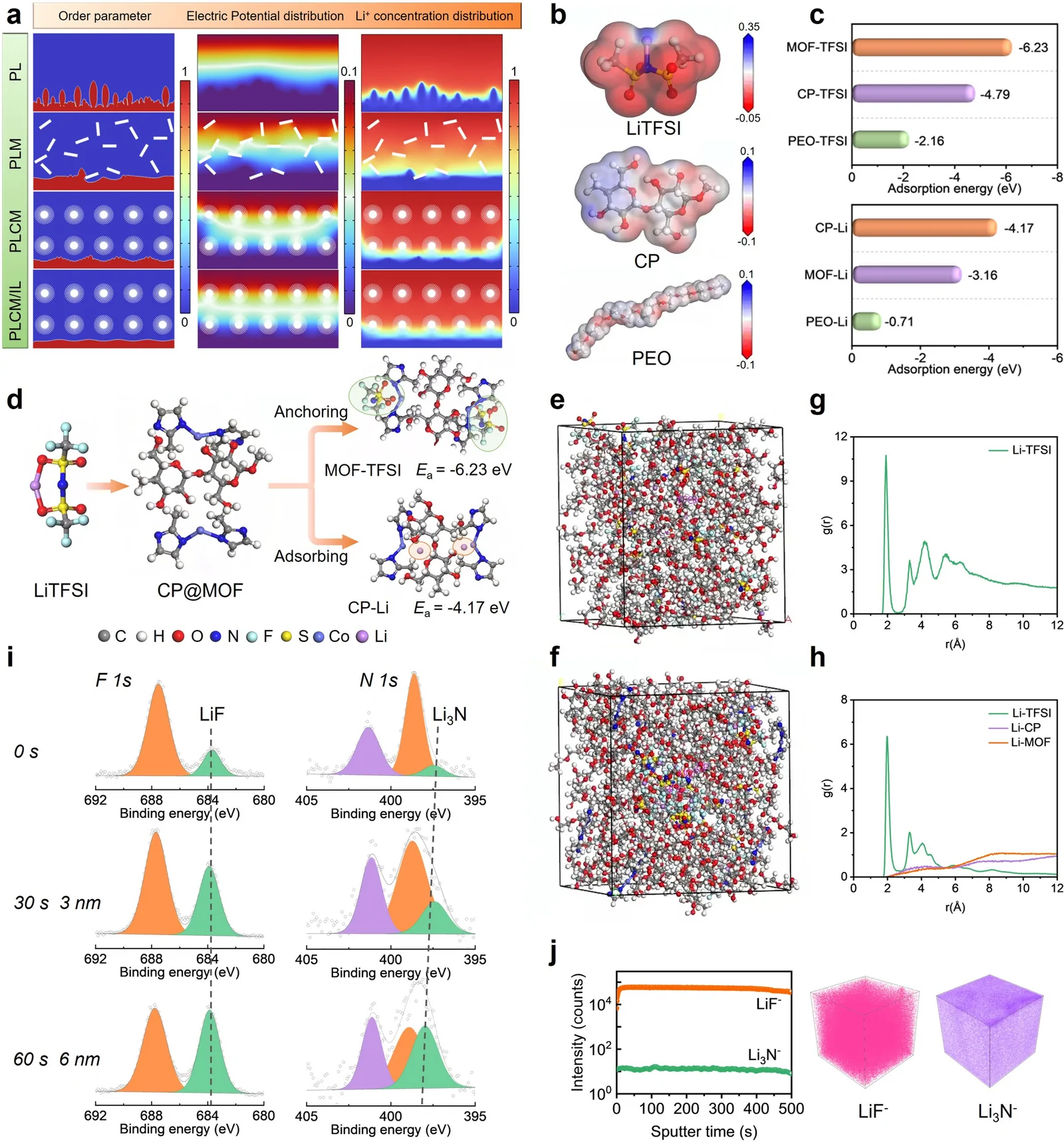
**a** Snapshots of the PL, PLM, PLCM and PLCM/IL electrolyte order parameters, electric potential distribution and Li^+^ concentration distribution as a function of time. **b** Molecular electrostatic potential (MESP) maps of LiTFSI, CP, and PEO. **c** Adsorption energies of dissociated TFSI^−^ and Li^+^ on PEO, CP, and MOF. **d** Optimized atomic model demonstrating the anchoring and absorbing effect of CP@MOF on LiTFSI. Snapshots based on MD simulation of **e** PL electrolyte and **f** PLCM/IL electrolyte system. Radial distribution functions g(r) of LiTFSI, Li-CP, and SN-MOF pairs calculated from MD simulation trajectories at 303.0 K for **g** PL electrolyte and **h** PLCM/IL electrolyte. **i** XPS spectra with depth profiles of the F 1*s* and N 1*s* elements and **j** TOF–SIMS depth profiles and the corresponding 3D spectra of LiF^−^ and Li_3_N^−^ on the PLCM/IL electrolyte surface after 100 h of cycling

To investigate the enhancement mechanisms of different electrolyte systems on interfacial stability, phase-field simulations were conducted using COMSOL Multiphysics to examine the influence of electrolyte support structures on lithium dendrite growth behavior (Fig. 4a). Figure S17 shows the temporal evolution of the electrolyte order parameter (*φ*): uncontrolled dendrite growth was observed in the PL system, the PLM system exhibited partially enhanced dendrite suppression, while in the PLCM and PLCM/IL composite electrolytes incorporating the CP@MOF network, dendrite growth was significantly slowed, and the morphology of the lithium deposition layer became more uniform and flatter. Simulation results of the electric potential and Li⁺ concentration distribution (Figs. S18 and S19) indicated that higher electric potential and Li^+^ concentration gradients existed at the dendrite tip regions in PL, driving accelerated dendrite growth. PLM showed slight improvement, but the variations in electric potential and Li^+^ concentration gradients were significantly smaller in PLCM and PLCM/IL. This suggests that the CP@MOF network not only enhances the mechanical strength of the polymer matrix to suppress dendrite penetration but also constructs efficient Li^+^ transport channels, promoting uniform lithium deposition. Finite element method (FEM) simulations were further employed to analyze the distribution of anions (TFSI⁻), Li^+^, and electric potential during deposition, resolving the dynamic interfacial evolution (Fig. S20). Driven by the applied electric field, anions and cations migrate through the electrolyte and accumulate on opposite sides of the electrode/electrolyte interface. The PL matrix failed to effectively alleviate this ionic distribution imbalance, leading to accumulated TFSI⁻ and Li^+^ concentrations and a significant potential difference at the interface. In contrast, the CP@MOF 3D network significantly reduced the TFSI⁻/ Li^+^ concentration gradient and the corresponding potential gradient at the interface. This is attributed to the higher *t*_Li_^+^ in PLCM and PLCM/IL electrolytes, effectively mitigating ionic concentration polarization and potential differences near the interface, thereby delaying dendrite growth and promoting uniform lithium deposition [46]. To reveal the interaction mechanism between CP@MOF and LiTFSI in the PLCM/IL electrolyte, density functional theory (DFT) calculations were performed. Electrostatic potential analysis of LiTFSI, CP, and PEO (Fig. 4b) showed that red regions (low electron cloud density, electrophilic) are more likely to gain electrons, while blue regions (nucleophilic) show the opposite tendency. Calculations of adsorption energies after structural optimization (Figs. 4c and S21) revealed that the adsorption energy of MOF for TFSI⁻ (− 6.23 eV) was significantly higher than that of CP (− 4.79 eV) and PEO (− 2.16 eV) for LiTFSI, confirming the strong affinity of MOF for TFSI⁻. Furthermore, CP exhibited the highest adsorption energy for dissociated Li^+^ (− 4.17 eV), indicating that dissociated Li⁺ is more readily absorbed by CP, forming Li^+^-rich region and creating additional pathways for Li^+^ transport, thereby enhancing ionic conductivity. Figure 4d schematically illustrates that MOF capture TFSI⁻ anions, restricting their movement and enabling efficient Li^+^ transport within the CP network, resulting in excellent electrochemical performance. Furthermore, molecular dynamics (MD) simulations reveal that the introduction of CP@MOF not only results in a more uniform spatial distribution of ions in the electrolyte (PLCM/IL) (Fig. 4e, f), but also significantly reduces the intensity of the characteristic peak (1.9 Å) in the radial distribution function (RDF) of Li⁺ and TFSI⁻ (Fig. 4g, h). This confirms that the CP@MOF structure effectively promotes LiTFSI dissociation, thereby increasing the concentration of free Li⁺ in the system [47].

To elucidate the chemical composition of the electrolyte/Li metal interface, the surface of the electrolyte from a Li symmetric cell after 100 cycles was analyzed by X-ray photoelectron spectroscopy (XPS). As shown in Fig. S22, a higher content of Li_3_N and LiF was detected in the PLCM/IL electrolyte. Further XPS depth profiling was performed on the cycled PLCM/electrolyte interface (Fig. 4i). The results show that the intensities of the characteristic peaks at 683.7 eV (LiF) and 397.8 eV (Li_3_N) gradually increased with increasing sputtering depth, indicating the enrichment and gradient distribution of these two components in the interfacial region. Li_3_N, as a fast ion conductor, significantly enhances the ionic conductivity of the SEI and reduces the interfacial impedance; while LiF, due to its low electronic conductivity and intrinsic electrochemical stability, effectively suppresses the penetration of Li dendrites into the electrolyte and the occurrence of side reactions. In addition, time-of-flight secondary ion mass spectrometry (TOF–SIMS) depth profiling was conducted on the PLCM/IL electrolyte interface. The elemental distribution curves and 3D reconstructed signals showed significant secondary ion intensities for LiF^−^ and LiN^−^ (Fig. 4j), which is consistent with the XPS results, collectively verifying the successful construction of a LiF/Li_3_N rich interface. The above results indicate that the CP@MOF 3D network efficiently adsorbs and anchors TFSI⁻ anions, restricting their migration and promoting the rapid transport of Li^+^ along the cellulose skeleton. Meanwhile, the strong electron-donating ability of the Lewis acid metal sites in the MOF facilitates the formation of Li_3_N [23], and the introduction of ILs promotes the in situ formation of LiF on the Li metal surface. The synergistic effect of the two guides the construction of a stable and uniform SEI layer, significantly promoting uniform Li^+^ deposition and effectively suppressing dendrite growth [48].

**Fig. 5:**
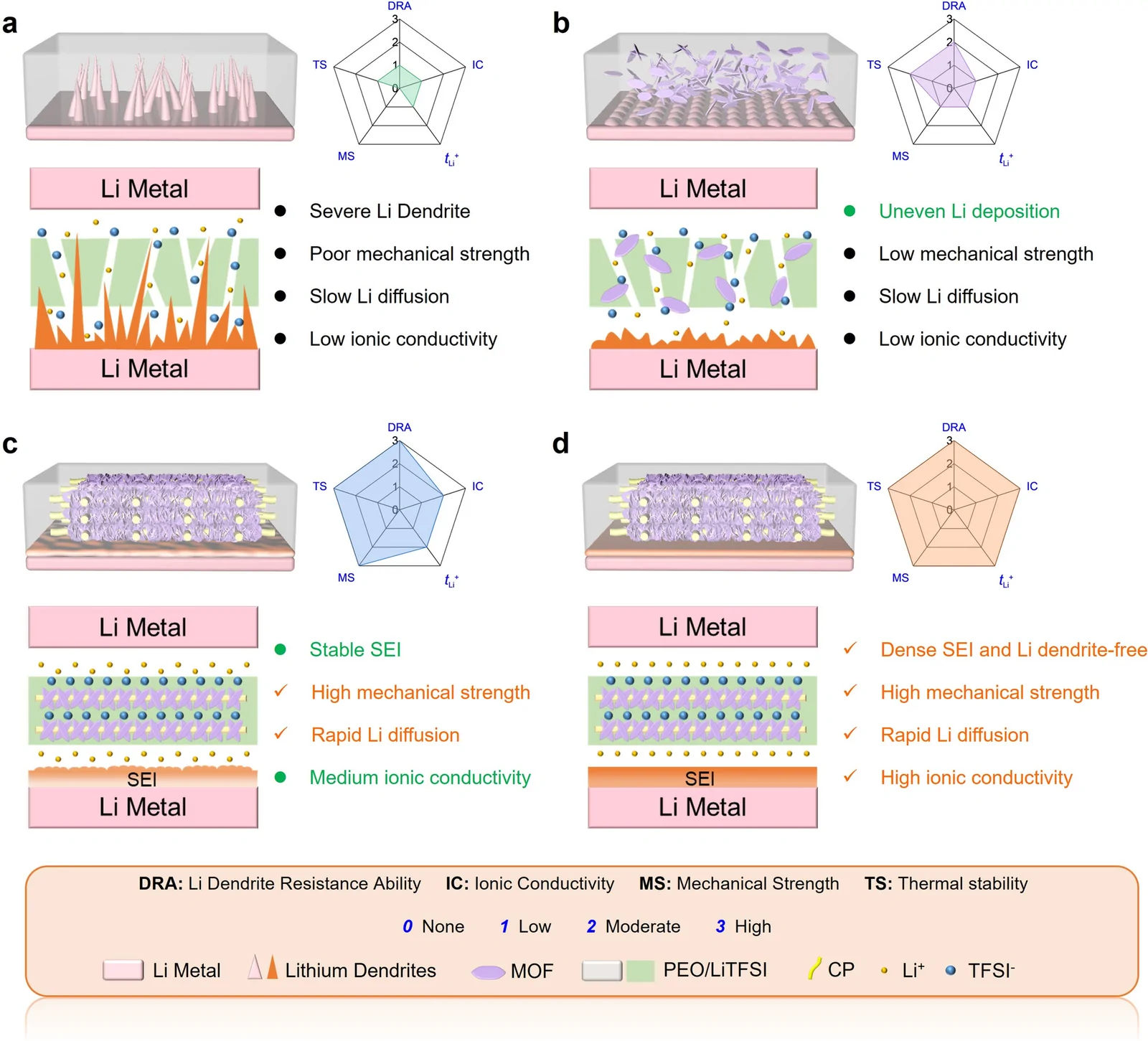
Schematic illustration and comparative hostile radar plots of the performance of SEI films and Li dendrites grown after cycling of **a** PL, **b** PLM, **c** PLCM, and **d** PLCM/IL electrolytes in Li symmetric cells

Based on the comprehensive analysis of the theoretical calculations and experimental results, the interfacial properties between four different electrolytes (PL, PLM, PLCM, and PLCM/IL) and the Li anode were systematically evaluated. As depicted in Fig. 5a, the PL polymer electrolyte, due to its inadequate mechanical properties, exhibits uncontrolled dendrite growth and fails to form an effective SEI. In comparison, the incorporation of MOF nanosheets slightly suppresses Li dendrite formation (Fig. 5b). However, the discrete distribution of MOF nanosheets is insufficient to construct a continuous network capable of effectively resisting dendrite propagation. Introducing a CP@MOF composite 3D network into the PL matrix significantly enhances the mechanical modulus of the electrolyte, thereby providing mechanical resistance against Li dendrite penetration. Concurrently, the in situ self-assembled MOF structure facilitates anion anchoring, which further promotes uniform Li^+^ deposition from an electrochemical perspective (Fig. 5c). Moreover, the introduction of ILs in the PLCM/IL electrolyte promotes the formation of a more stable and compositionally richer SEI layer, substantially improving interfacial stability (Fig. 5d). As a result, the Li|PLCM/IL|Li symmetric cell demonstrates exceptional cycling stability. In summary, the CP@MOF network serves as an efficient 3D ion transport pathway within the polymer matrix. Its superior interfacial stability combined with low interfacial impedance significantly facilitates Li⁺ transport, thereby enhancing the overall electrochemical performance of the battery.

### Electrochemical Performance of SSLMBs

To verify the feasibility for practical battery applications, SSLMBs were assembled using four different electrolytes with LFP cathode and Li anode, respectively. As shown in Fig. S23, the LFP|PLCM/IL|Li battery employing the PLCM/IL electrolyte exhibited the highest discharge specific capacity at various rates, particularly maintaining stable capacity output even at high rates. Additionally, this battery displayed smooth charge/discharge voltage profiles at different rates (Fig. S24), indicating good electrode–electrolyte interfacial contact, which facilitates ion transport. The long-term cycling performance of different LFP|Li full cells assembled with various electrolytes was further compared. The LFP|PL|Li battery not only showed significant fluctuations in the initial charge/discharge curves (Fig. S25a), but also experienced rapid specific capacity fading and poor cycling stability at 0.2 C (Fig. S25b). This instability may originate from the uncontrolled growth of Li dendrites. In contrast, batteries employing PLCM and PLCM/IL electrolytes formed abundant SEI layers at the Li anode interface, thereby exhibiting excellent cycling stability at 0.2 C. Furthermore, the PLCM/IL electrolyte, due to its superior mechanical properties and low interfacial impedance (Fig. S26), ensured efficient Li⁺ transport in SSLMBs even at higher rates. Even after 1000 cycles at 0.5 C, this battery maintained a high capacity retention of 94.6% and a stable coulombic efficiency above 98% (Fig. S25c), significantly outperforming other recently reported SSLMBs (Fig. S27, Table S3) [33, 34, 39, 40, 47–50]. The structural characterization and performance analysis above demonstrate that the PLCM/IL composite electrolyte system constructed in this study successfully achieved multi-scale structural regulation: at the molecular scale, the ion-sieving effect of MOF nanopores optimized carrier transport; at the mesoscale, an oriented nanosheet network established fast ion channels; at the macroscale, a 3D continuous structure ensured mechanical integrity and interfacial stability. This multi-level structural design strategy provides a new material system construction approach for developing high-performance SSLMBs.

**Fig. 6:**
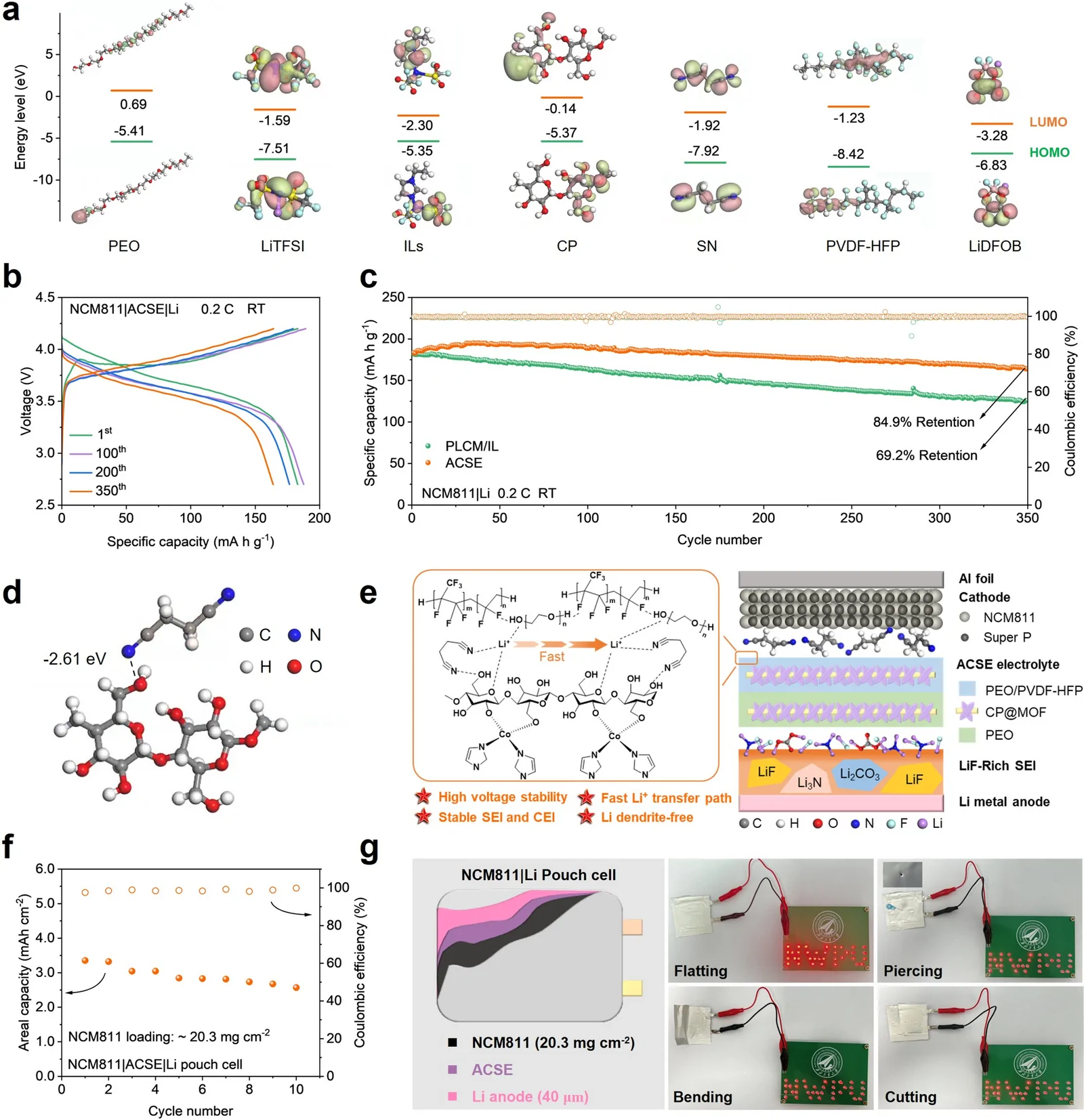
**a** Molecular structure and the calculated HOMO/LUMO energy levels (eV) of PEO, LiTFSI, ILs, CP, SN, PVDF-HFP, and LiDFOB. **b** Charge and discharge voltage profiles and **c** long-term cycling at 0.2 C of NCM811|ACSE|Li cells. **d** Binding energy between CP-OH and SN–C≡N calculated by DFT methods. **e** Schematic of Li^+^ transport in NCM811|ACSE|Li cells. **f** Cycle performance of the NCM811|ACSE|Li pouch full cell. **g** Photographs of solid-state pouch Li metal cell based on ACSE composite solid electrolyte showing well working at flat, bent, pierced, and cut states

The compatibility of the PLCM/IL electrolyte with high-voltage cathodes was further evaluated. As shown in Fig. S28, the NCM|PLCM/IL|Li battery exhibited a capacity retention of only 50% after 350 cycles, indicating significant capacity fading and limitations in high-voltage applications. Previous experiments confirmed the excellent interfacial compatibility of PLCM/IL with Li metal anodes. To enhance the performance of high-voltage batteries, an asymmetric solid composite electrolyte (ACSE) was designed, with its structure illustrated in Fig. S29. This ACSE retains the PLCM/IL layer facing the Li anode side while introducing a modified layer containing PVDF-HFP and SN facing the high-voltage NCM811 cathode side to improve interfacial stability. The SEM surface images on both sides, together with the corresponding EDS mapping results collectively demonstrate a good infiltration and integration among the various components (Figs. S30 and S31). Density functional theory (DFT) calculations were employed to determine the highest occupied molecular orbital (HOMO) and lowest unoccupied molecular orbital (LUMO) energy levels to evaluate their respective redox properties. As shown in Fig. 6a, PEO possesses a higher HOMO energy level (− 5.41 eV), indicating its relative instability. In contrast to PEO, PVDF-HFP exhibited a lower HOMO energy (− 8.42 eV), signifying enhanced oxidative stability at high potentials. The LUMO energy levels were used to assess electrolyte–electrode interfacial compatibility. The LUMO energy levels of LiTFSI, ILs, CP, SN, PVDF-HFP, and LiDFOB (− 1.59, − 2.30, − 0.14, − 1.92, − 1.23, and − 3.28 eV) were all significantly lower than that of the PEO matrix (− 0.69 eV). This demonstrates that the components introduced in the ACSE effectively improve interfacial compatibility, facilitating the formation of a stable interfacial layer [51]. This asymmetric design, by optimizing the compatibility and stability of each component at their respective electrode interfaces, is critical for ensuring the stable operation of high-voltage SSLMBs.

To verify the practical feasibility of ACSE, its electrochemical performance was first evaluated. Benefiting from the further reduction in crystallinity and improved high-voltage tolerance of the PEO matrix by the SN plasticizer [31, 52], the ionic conductivity (4.39 × 10^–4^ S cm^−1^, 30 °C) and electrochemical stability window (5.08 V) of ACSE were significantly enhanced (Fig. S32). ACSE exhibits superior ionic conductivity and electrochemical stability windows compared to most recently reported composite solid-state electrolytes (Fig. S33). Simultaneously, the *t*_Li_^+^ slightly increased (0.68, Fig. S34), indicating that ion transport remains efficient in the asymmetric structure. Secondly, the LFP|Li battery assembled with ACSE exhibited excellent rate capability (Fig. S35) and stable long-term cycling performance (Fig. S36). Meanwhile, the interfacial impedance values of the battery remained at a low level both before and after cycling (Fig. S37). Notably, the LFP|ACSE|Li full cell maintained a capacity retention rate of 82.9% after 500 cycles at 1.0 C (Fig. S38). Finally, high-voltage SSLMBs were assembled using ACSE as the electrolyte, NCM811 as the cathode, and Li metal as the anode. Tests revealed that the NCM811|ACSE|Li battery not only displayed more stable charge/discharge voltage profiles (Fig. 6b) and consistent impedance changes (Fig. S39) at 0.2 C, but also delivered a discharge specific capacity of 164.7 mAh g^−1^ after 350 cycles with a capacity retention rate of 84.9% (Fig. 6c), significantly outperforming the NCM811|PLCM/IL|Li battery. Besides, rate performance tests of NCM811|ACSE|Li batteries demonstrate that their specific capacity remains above 100 mAh g⁻^1^ even at high rates of 1.0 C (Fig. S40). To elucidate the intrinsic mechanism behind the improved cycling performance of the high-voltage SSLMBs, the SSLMBs after 50 cycles were disassembled. XPS analysis of the ACSE/NCM811 interface was conducted to characterize the composition of the cathode/electrolyte interface (CEI) layer (Fig. S41). Comparison of spectra before and after cycling revealed the formation of a CEI layer rich in LiF and Li_3_N at the cathode/electrolyte interface. Additionally, XPS spectra further confirm the formation of a boron-containing CEI induced by LiDFOB. This interfacial layer effectively suppresses electrolyte decomposition and simultaneously mitigates lattice oxygen loss and cathode structural degradation through strong bonding interactions between boron-enriched functional groups (B–O/B–F bonds) and lattice oxygen atoms on the cathode surface [53]. Simultaneously, TOF–SIMS 3D depth profiles detected Li_3_N^−^, LiF^−^, and B^−^ fragment ions, confirming the formation of a spatially continuous interfacial layer, consistent with XPS analysis (Fig. S42). The presence of −C≡N groups derived from SN was detected, confirming that SN participated in the interfacial reaction. The binding energy between structurally optimized CP and SN was further calculated (Fig. 6d). The binding energy between the −C≡N group in SN and the −CH_2_–OH group in CP was − 2.61 eV, indicating an adsorption effect of CP molecules on SN, thereby inhibiting the shuttle of SN and preventing corrosion of the Li anode by SN. Figure 6e shows a schematic diagram of the NCM811|ACSE|Li battery structure. The synergistic effects of the ACSE components manifest four major advantages: high-voltage stability; rapid ion transport pathways; stable SEI and CEI layers; and a dendrite-free deposition interface (Fig. S43).

To evaluate the practical feasibility of ACSE, pouch cells were assembled using high-loading NCM811 cathodes (~ 20.3 mg cm^−2^) and their electrochemical performance was evaluated. As shown in Fig. 6f and Table S4, the pouch cell delivered an areal capacity of 3.35 mAh cm^−2^, with an energy density of 337.9 Wh kg^−1^/ 711.7 Wh L^−1^. Meanwhile, NCM811|ACSE|Li pouch cell maintain stable cycle performance even under high-temperature conditions (Fig. S44). Impressively, the pouch cell maintained functionality under folding, bending, and even cutting conditions (Fig. 6g), demonstrating exceptional safety and stability of the ACSE electrolyte in SSLMBs.

## Conclusions

In summary, an asymmetric composite solid-state electrolyte (ACSE) film was designed by in situ self-assembling 2D MOF nanosheets on a 3D cellulose scaffold to construct a CP@MOF 3D network, which was then integrated with a polymer matrix to simultaneously achieve enhancement of mechanical strength and interfacial stability. Additionally, the introduction of IL and SN enhanced polymer chain segment mobility, thereby promoting Li migration and diffusion. Consequently, the ACSE electrolyte delivered excellent ionic conductivity (4.39 × 10^–4^ S cm^−1^, high Li^+^ transference number (0.68), broad electrochemical stability window (5.08 V), and significantly improved Young's modulus (1.19 GPa). Theoretical calculations and experimental evidence confirmed that the high-modulus electrolyte forms a mechanical barrier to suppress Li dendrite growth at the mechanical level, while the 3D CP@MOF network concurrently facilitates Li⁺ migration and anchors TFSI⁻ anions to regulate uniform Li⁺ deposition at the electrochemical level. Benefiting from mechano-electrochemical synergistic coupling, Li symmetric cells achieved stable plating/stripping for over 5000 h. Notably, stable SEI and CEI interphases formed at interfaces due to unique structural and compositional integration, ensuring stable cycling of high-voltage cathodes. More impressively, NCM811|ACSE|Li pouch cells attained energy densities of 337.9 Wh kg^−1^ and 711.7 Wh L^−1^. The maintained functionality under extreme conditions demonstrates practical application potential. This work establishes a novel synergistic paradigm of high modulus and interfacial stability in ACSE design, providing a universal strategy for highly safe SSLMBs.

## Supplementary Information

Below is the link to the electronic supplementary material.

Supplementary file1 (DOCX 11127 KB)
