# Supplementary material for: Mechano-Electrochemical Synergy in Cellulose@MOF Scaffold-Based Asymmetric Electrolyte for Stable Solid-State Lithium Metal Batteries
Source: Nanomicro Lett. 2026 Jan 15;18:208. doi: 10.1007/s40820-025-02039-x (PMC12804580; doi:10.1007/s40820-025-02039-x)
Supplement: Supplementary file 1 — Supplementary file1 (DOCX 11127 KB) [file 40820_2025_2039_MOESM1_ESM.docx]

**Supporting Information**

**Mechano-Electrochemical Synergy in Cellulose@MOF Scaffold-Based Asymmetric Electrolyte for Stable Solid-state Lithium Metal Batteries**

*Wanqing Fan^[a]^, Xuetao Shi^[a]^, Ying Huang^*[a]^, Kaihang She^[a]^, Bowei Song^[a]^, and Zheng Zhang^*[b]^*

^[^*^a^*^]^ Dr. W. Fan, Prof. X. Shi, Prof. Y. Huang, Dr. K. She, Dr. B. Song

The MOE Key Laboratory of Material Physics and Chemistry under Extraordinary Conditions, Ministry of Education, School of Chemistry and Chemical Engineering, Northwestern Polytechnical University, Xi’an 710072, China.

E-mail: yingh@nwpu.edu.cn

^[^*^b^*^]^ Dr. Z. Zhang

State Key Laboratory of Efficient Production of Forest Resources, Beijing Key Laboratory of Lignocellulosic Chemistry, Beijing Forestry University, Beijing 100083, China

E-mail: zhengzhang0527@163.com

**Table of Contents**

[**1 Experimental 2**](#_Toc213254283)

[**2 Figure S1-figure S44 7**](#_Toc213254284)

[**3 Table S1-S4 25**](#_Toc213254285)

[**4 Reference 28**](#_Toc213254286)

# 1 Experimental

**1.1 Preparation of materials**

***Materials:*** Cellulose paper barrier film (CP, NKK-MPF30AC-100, Canrd), Co(NO_3_)_2_·6H_2_O (99%, Aladdin), 2-methylimidazole (2-Mlm, 99%, Aladdin), poly(ethylene oxide) (PEO, M_w_=600,000, Aladdin), lithium bis(trifluoromethane)sulfonylimide salt (LiTFSI, 99%, Aladdin), 1-Ethyl-3- methylimidazolium bis(trif-luoromethanesulfonyl) imide (ionic liquids, ILs, 99%, Aladdin), Poly(vinylidene fluoride- co -hexafluoropropylene) (PVDF-HFP, M_w_=400,000 , Sigma), Lithium borate difluorooxalate (LiDFOB, 99%, Aladdin), succinonitrile (SN, 99%, Aladdin), acetonitrile (AR, 99%, Aladdin), n-methyl-2-pyrrolidone (NMP, 99.5%, Aladdin).

***Preparation of CP@MOF films*** ***grown in situ:*** Typically, 0.58 g of Co(NO_3_)_2_·6H_2_O was dissolved in 40 mL of water to form solution A, and 1.31 g of 2-Mlm was dissolved in 40 mL of water to form solution B. Each of them was stirred for 20 min, and then B was rapidly poured into A, and stirred for 5 min. The CP was placed at the bottom of the solution, and then left to stand for 2 h. The other side of the cellulose paper was ultrasonically cleaned with ethanol and then the cellulose paper was subjected to secondary deposition, and the above steps were repeated. Subsequently, the in situ synthesized CP@MOF films were ultrasonically cleaned in ethanol and vacuum dried at 60 ℃ for 12 h for subsequent use.

***Preparation of PHML/IL electrolyte:*** PEO and LiTFSI (EO:Li=15:1) were dissolved in acetonitrile, 10wt% IL was added and stirred continuously for 12 h, then half of it was poured into a polytetrafluoroethylene (PTFE) mould, which flowed and was poured into a CP@MOF film. The other half of the mixture was then poured in. After waiting for the solvent to dry in a vacuum drying tray, the PEO/LiTFSI/CP@MOF composite solid electrolyte film was obtained, named PLCM/IL. To set up a control group, a PEO and LiTFSI polymer matrix, named PL, was prepared. 20 wt% of MOF particles were dispersed in the polymer matrix to obtain PLM electrolyte films. In addition, a PEO and LiTFSI polymer matrix named PLCM was cast in CP@MOF films.

***Preparation of asymmetric electrolyte (ACSE):*** PEO and LiTFSI (EO:Li=15:1) were dissolved in acetonitrile, 10wt% ILs was added with continuous stirring for 12 h and then poured into PTFE moulds and then into CP@MOF films. Another PVDF-HFP was dissolved in DMF, PEO and LiTFSI with LiDFOB (the mass of LiTFSI remains unchanged, with a mass ratio of LiODFB to LiTFSI at 1:2). dissolved in acetonitrile (1:4 mass ratio of PVDF-HFP: PEO), followed by the addition of 10 wt% of SN, all mixed for 12 h with continuous stirring and then poured onto the other side of the CP@MOF film. The film was dried in a vacuum drying dish for 24 h and then transferred to a vacuum drying oven at 60 °C for 24 h. Asymmetric composite solid electrolyte (ACSE) films were obtained.

***Preparation of cathode materials:*** LiFePO_4_ (LFP) or LiNi_0.8_Mn_0.1_Co_0.1_O_2_ (NCM811, Canrd Technology Co. Ltd), Super P, and PVDF (polyvinylidene fluoride) were milled in NMP solution in a mass ratio of 80:10:10, and then cast on aluminum foil. The cathode film was vacuum dried at 80 °C for 24 h and then perforated into pellets with a diameter of 10 mm, which were subsequently used for coin cell assembly. Unless otherwise specified, the active material weight of cathode was about 2.5 mg cm^-2^.

**1.2 Characterizations**

The crystal structure was measured with an x-ray diffractometer (XRD, Bruker D8 Advance). Scanning electron microscopy (SEM, FEI Verios G4) and high-resolution transmission electron microscopy (TEM, Talos F200X TEM) were used to observe the structural morphology and matched energy dispersive spectroscopy (EDS) was used to determine the elemental mapping of selected regions. The N_2_ adsorption/desorption isothermal was performed by the BELSORP-mini II (Macchik Bayer, Japan). Thermogravimetric (TGA, Mettler Model Q50) analyses were performed in N_2_ with a heating rate of 10°C min^-1^. Differential scanning calorimetry (DSC, Mettler-Toledo) tests were performed in N_2_ with a heating rate of 5 °C min^-1^. Stress-strain curves were performed using a sample size of 10 cm×2 cm×32.5μm and measured using an Instron Bluehill LE with a constant velocity of 1 mm min^-1^. To evaluate the mechanical robustness of the CSEs, puncture resistance tests were conducted using Instron Bluehill LE. A steel needle with a diameter of 0.5 mm was driven at a constant crosshead speed of 0.5 mm/min towards a clamped CSE sample with an exposed diameter of 15 mm. The test was automatically terminated upon complete penetration of the sample. The surface composition and chemical composition of the samples were analyzed using x-ray photoelectron spectroscopy (XPS, Kratos AXIS Ultra DLD), and all the binding energies obtained in XPS spectra were calibrated using the C 1 s peak at 284.6 eV. TOF-SIMS (IONTOF GmbH, Münster, Germany) was conducted on a TOF-SIMS spectrometer attached to the SEM. The TOF-SIMS measurements were conducted in negative modes.

**1.3 Cell assembly and Electrochemical measurements**

***Cell assembly:*** The prepared PL, PLM, PLCM, PLCM/IL and ACSE membranes were cut into 17 mm diameter discs and placed in a glove box filled with Ar_2_ atmosphere. Solid-state lithium metal batteries (SSLMBs) are prepared by using LFP/NCM811|CSEs|Li metal. Notably, the prepared asymmetric ACSE electrolyte contained PVDF-HFP and SN on the side toward the cathode and ILs on the side toward the Li metal anode. Unless otherwise specified, the active material weight of cathode was about 2.5 mg cm^−2^. To enable the SSLMBs to charge and discharge at room temperature, 2 μL cm^-2^ liquid electrolyte (1.0 mol L^-1^ LiPF_6_/ethylene carbonate(EC):diethylcarbonate(DEC): ethylmethylcarbonat(EMC) = 1:1:1, by volume) was used to infiltrate the cathode side to enhance interfacial contact. The LPF|CSEs|Li SSLMBs were charged and discharged between 2.7~3.8 V. The NCM811|CSEs|Li SSLMBs were charged and discharged between 2.7~4.2 V. The long-term cycling tests of Li symmetric batteries were performed by galvanostatic charging and discharging at current densities/time of 0.2 mA cm^-2^ /0.5 h.

Ionic conductivity (IC), linear sweep voltammetry (LSV) and Li^+^ transference number (*t*_Li_^+^) were collected with a Gamry Interface 1010 electrochemical workstation.

***Ionic conductivity (IC):*** the CSEs was sandwiched between two stainless steel plates at temperatures from 30 to 80 °C and the frequency range was 0.1 to 10^6^ Hz (SS/CSEs/SS). The IC was calculated by the following formula.

$\sigma=\frac{t}{RA}$ Eq.S1

Where t represents the thickness of the CSEs, R represents the impedance, and A is the area of the stainless−steel (16 mm disc).

$\sigma_{T}=Aexp(\frac{-E_{a}}{RT})$ Eq.S2

Where σ_T_ is the ionic conductivity at different temperatures, E_a_ is the activation energy, T is the absolute temperature, and A is the pre−exponential factor.

***Electrochemical window:*** The LSV test was performed by assembling a Li/CSEs/SS cell at a scan rate of 0.5 mV s^−1^ from 1.0 to 6.0 V to evaluate the electrochemical stability of the CSEs to lithium metal (60 °C).

***Li^+^ transference number (t_Li_^+^):*** *t*_Li_^+^ was measured by the chronoamperometry and calculated according to following formula (60 °C).

$t_{{Li}^{+}}=\frac{I_{ss}(\Delta V-I_{0}R_{0})}{I_{0}(\Delta V-I_{ss}R_{ss})}$ Eq.S3

Where I_0_ is the initial current, I_SS_ is the steady−state current, R_0_ is the interface impedance before polarization, and R_SS_ is the interface impedance after polarization, ΔV is voltage bias (10 mV).

**1.4 DFT calculation**

The density functional theory (DFT) was used to perform the quantum chemistry calculations by Dmol3 module. The equilibriumstate structures with geometry optimization were performed by employing the three-parameter empirical formulation B3LYP. The electrostatic potential, binding energy and the highest occupied molecular orbital (HOMO) and lowest unoccupied molecular orbital (LUMO) were analyzed.

The adsorption energy (E_a_) of TFIS^−^ or Li^+^ to the PEO, CP and MOF is defined as the following:

$E_{a}=E\left( A-B \right)-[E\left( A \right)-E(B)]$ Eq.S4

where E_a_ is the total energy of the relaxed PEO/CP/MOF−TFSI/Li system and E(A) and E(B) are energies of the free-standing PEO/CP/MOF and the TFSI/Li with the same density.

**1.5 MD calculations**

To investigate the influence of CP@MOF on polymer-mixed systems, molecular dynamics (MD) simulations were employed to calculate the radial distribution functions (RDF) for both the PL and PLCM/IL systems. The PL system comprised 150 PEO segment molecules and 20 LiTFSI molecules, while the PLCM/IL system was supplemented with 10 MOF and 10 CP molecules. The components were respectively constructed into cubic simulation boxes. All MD simulations were carried out by Forcite module with COMPASS Ⅲ force field. Van der Waals and Coulomb interactions were respectively considered by atom based and Ewald methods with a cut-off value of 20 Å. Equations of motion were integrated with a time step of 1 fs. After energy minimization, each system was fully relaxed under periodic boundary conditions for 200 ps in the NPT (P = 1 atmosphere, T = 303.0 K) ensemble using the Nose thermostat and Berendsen barostat, which was long enough for system temperature, potential and total energy to get stable. After reaching equilibrium state, another 200 ps simulation under NVT ensemble was performed to extract trajectory and data for RDF calculation. The dynamic trajectory for each system was outputted at an interval of 1 ps.

**1.6 Simulation of Li dendrite evolution**

From the physical point of view, Li^+^ moves towards the electrode surface because of the overpotential divergence between the anode and cathode. As a result, Li^+^ is reduced to lithium atoms and deposits on the Li foil surface, inducing the formation and growth of Li dendrites. To simulate the complicated morphological evolution of Li dendrites regulated by solid electrolytes, a Phase-field model (PFM) has been developed. For simplicity, two reasonable assumptions are made: First, the solid fillers are extracted as solid particles uniformly distributed along the anode surface. Second, the impact of lithium dendrites on the solid fillers are ignored. The time-dependent evolution of Li electroplating is expressed by the following equations:


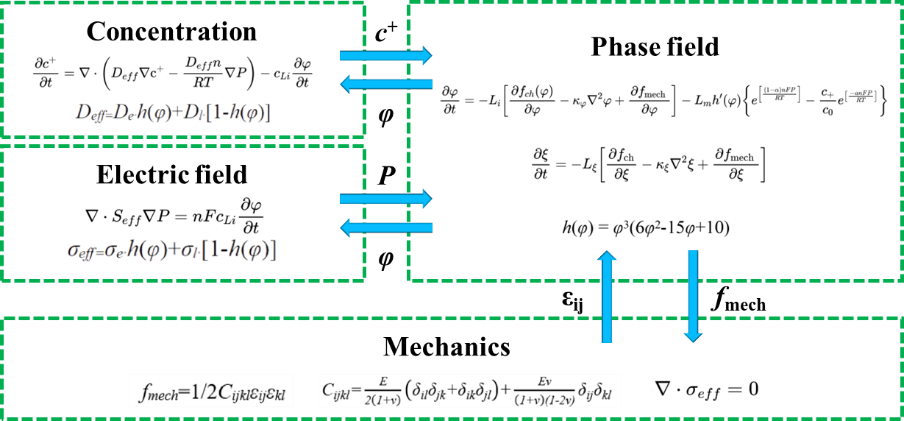


**1.7 Simulation of Li^+^ concentration and potential distribution**

The transport of Li^+^ stimulated by electric fields were imitated based on Electrostatic and Transport of Diluted Species modules combined in COMSOL Multiphysics. The physical model is governed by Nernst-Planck-Poisson equations:


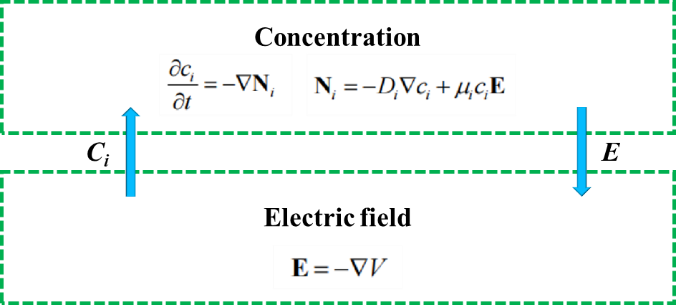


where V is the overpotential, E is the electric field, *D*_i_ is the diffusion coefficient of species i, *c*_i_ is the concentration of species i, *μ*_i_ is the ionic mobility of species i, and *N*_i_ is the flux vector of species i. As the driving force for Li^+^ transport, the overpotential difference between the anode and cathode was set as 0.01 V. The Li^+^ flux on the boundary, which resulted from the electrochemical reaction, was set as 0.01 mol m^-2^ s^-1^. The diffusion coefficient is derived from the ionic conductivity based on Nernst-Einstein equation. According to the Li^+^ transference number, anions were divided into two sets: one corresponds to the free anions with the normal diffusion coefficient, the other corresponds to the immobilized anions with the diffusion coefficient of 0.

# 2 Figure S1-figure S44


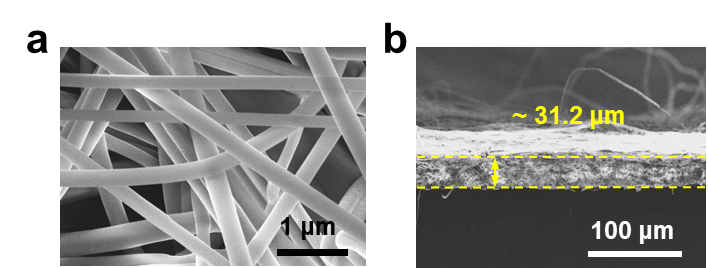


**Figure S1** SEM images of the (a) surface and (b) cross section of CP.


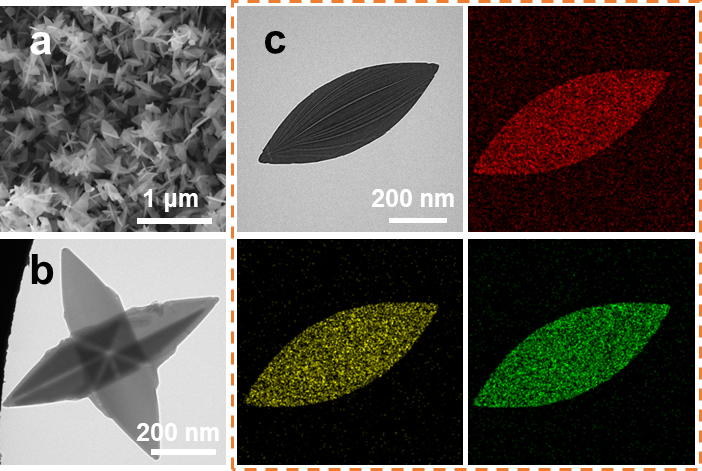


**Figure S2** (a) SEM image (b) TEM image and (c) EDS mapping of MOFs.


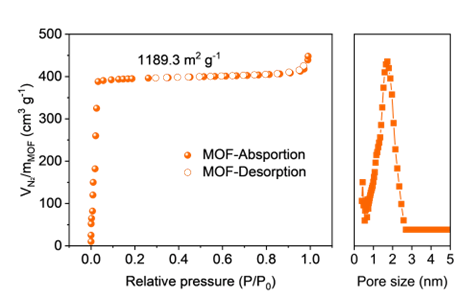


**Figure S3** The N_2_ adsorption/desorption curves of the MOFs, the right part is the corresponding pore size distribution curves.


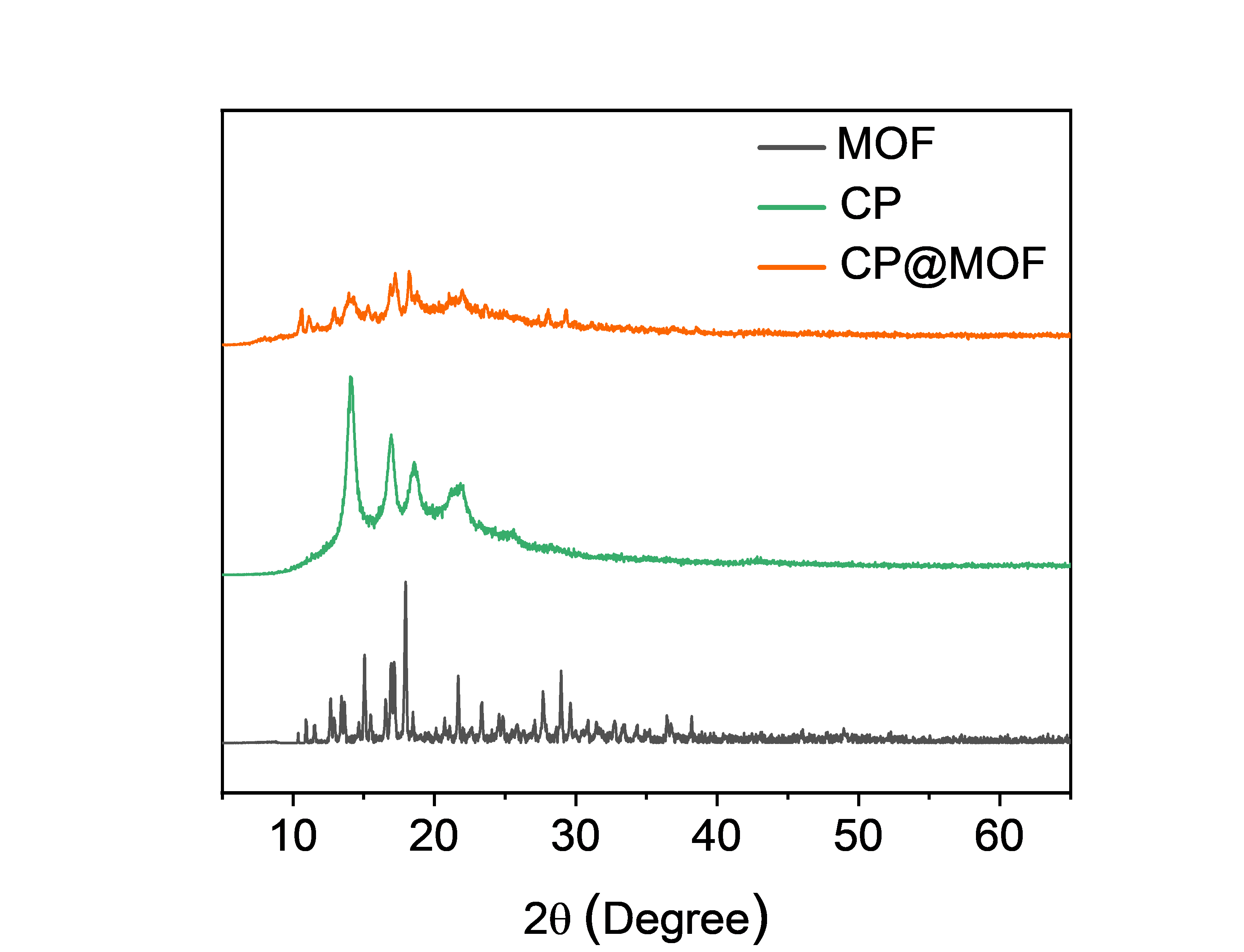


**Figure S4** XRD patterns of MOF, CP and CP@MOF.


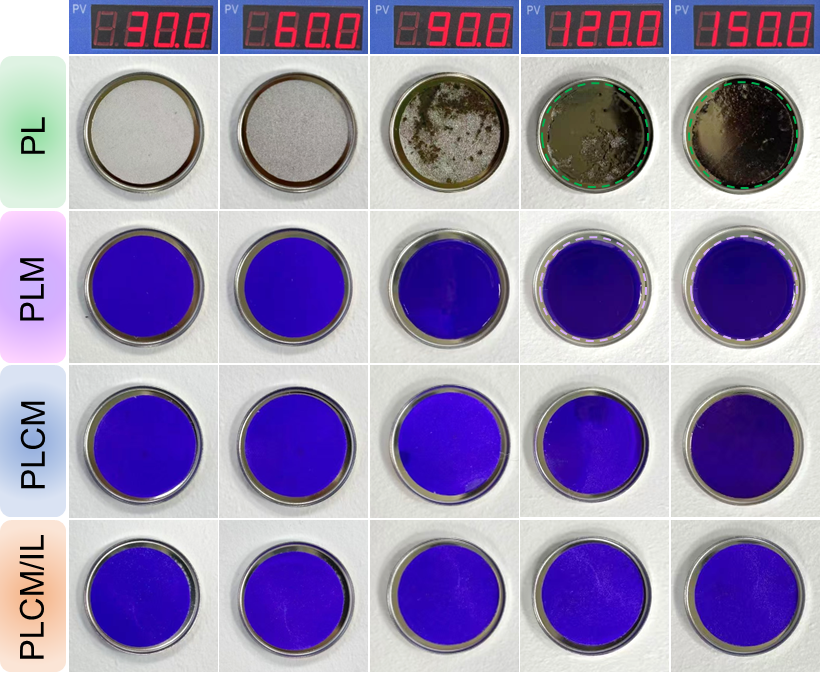


**Figure S5** The evolution of the electrolyte state with temperature.


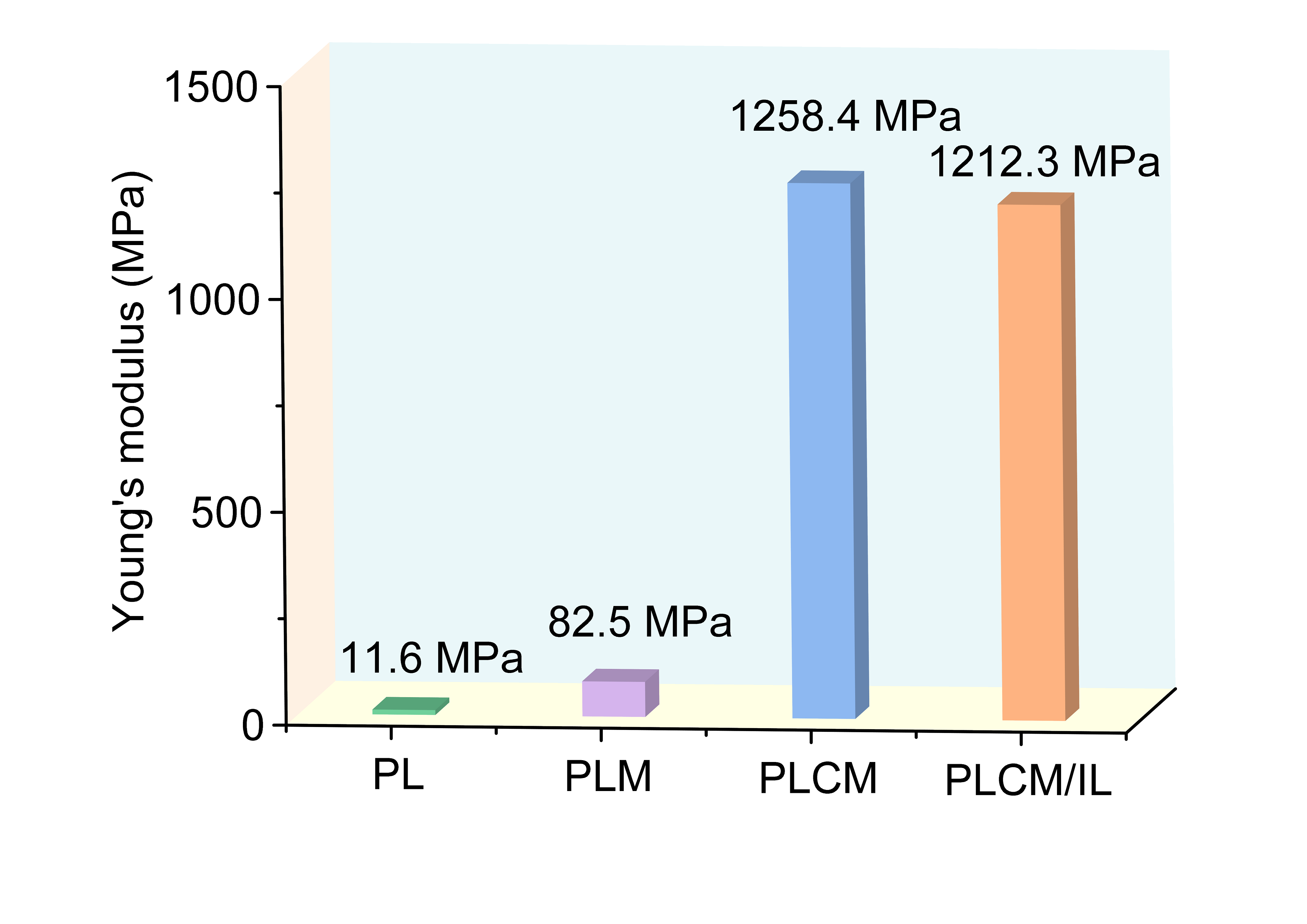


**Figure S6** Young's modulus of PL, PLM, PLCM and PLCM/IL.


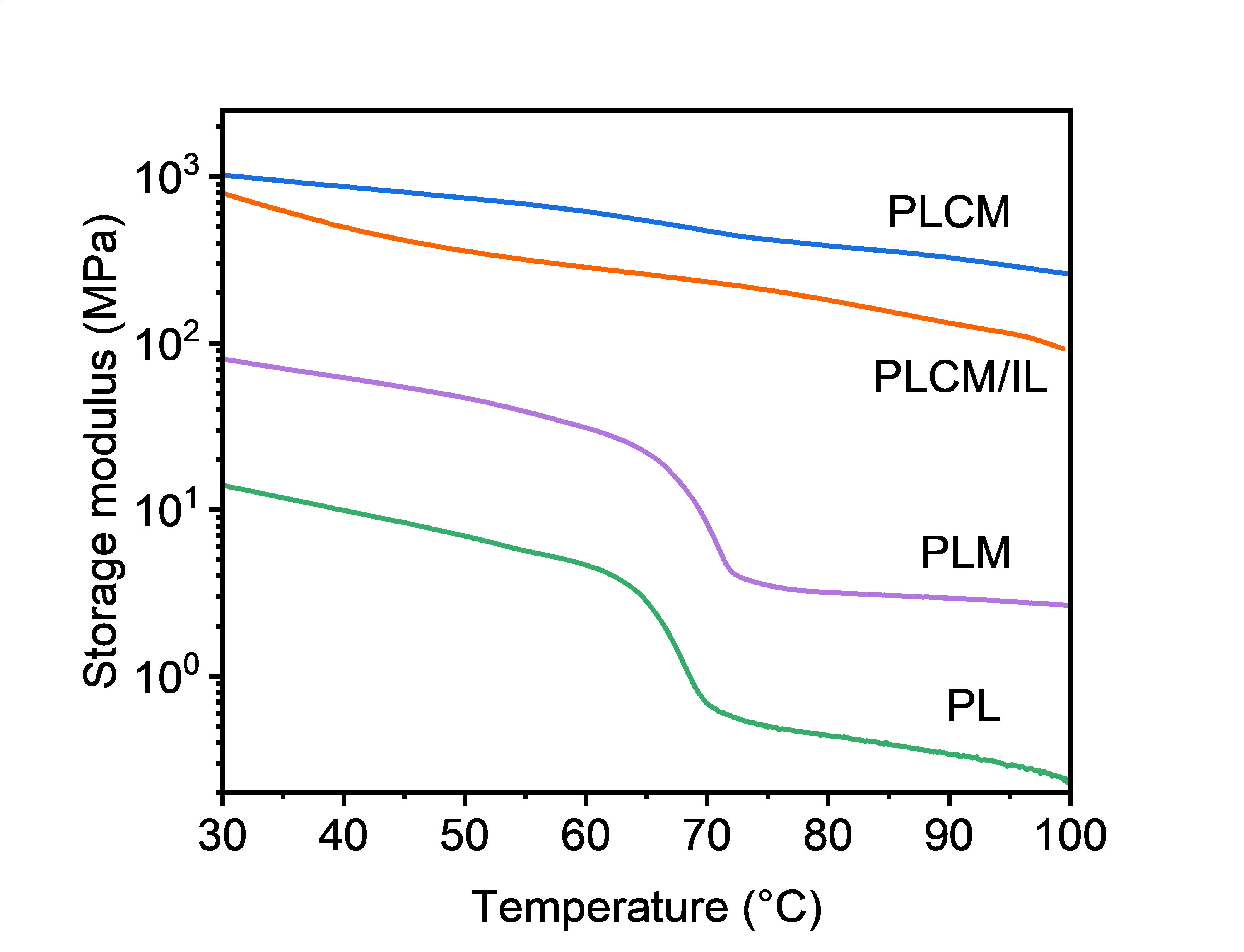


**Figure S7** Storage modulus as a function of temperature for PL, PLM, PLCM and PLCM/IL.


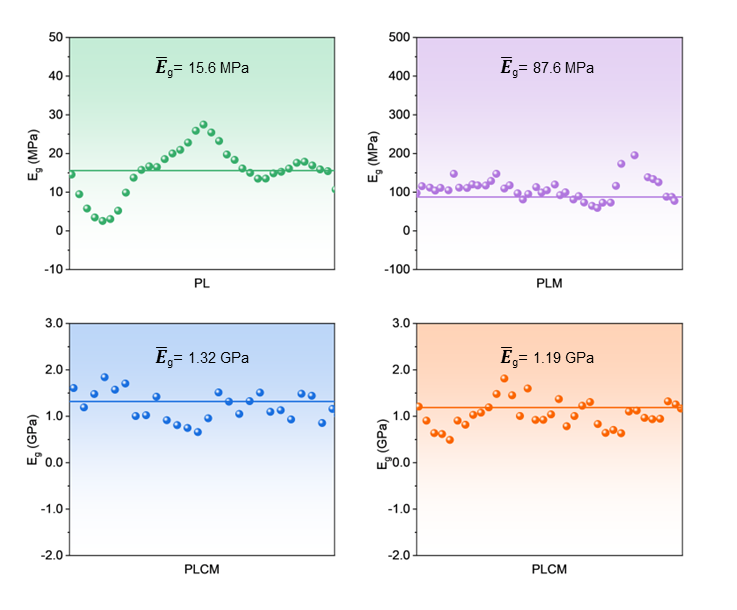


**Figure S8** The elastic modulus mapping of CSEs obtained by AFM.


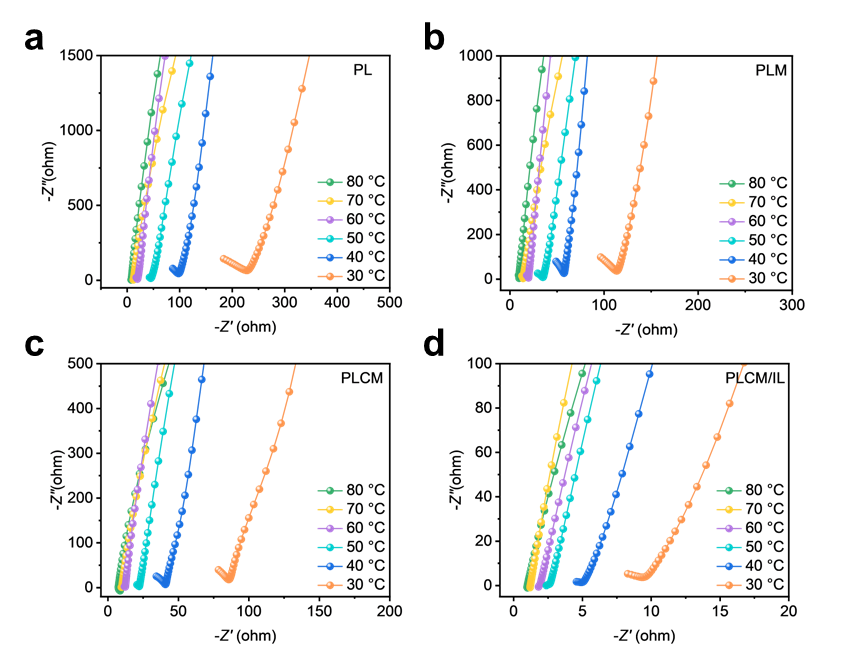


**Figure S9** Nyquist plots of (a) PL, (b) PLM, (c) PLCM and (d) PLCM/IL from 30 ℃ to 80 ℃.


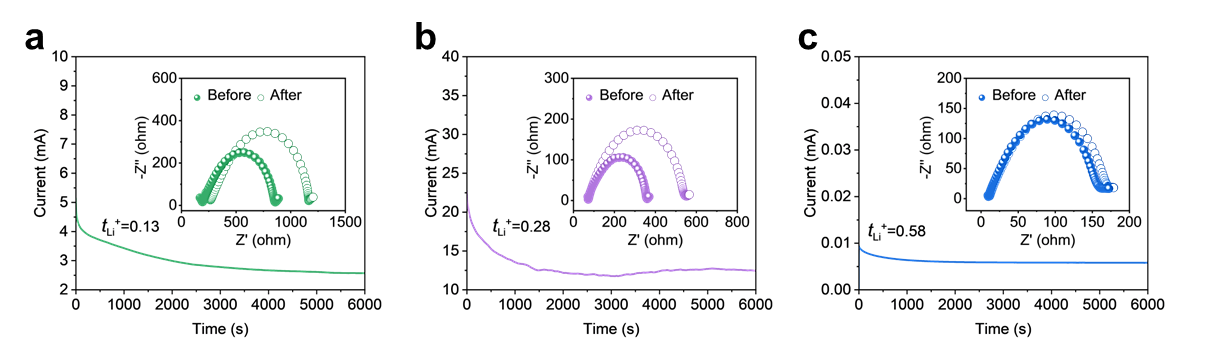


**Figure S10** Li^+^ transfer number of (a) PL, (b) PLM, and (c) PLCM.


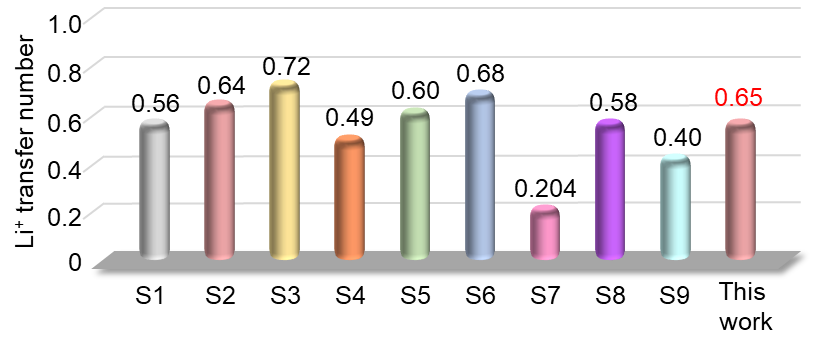


**Figure S11** Performance comparison between this work and other publications in terms of Li^+^ transfer number.[1-9]


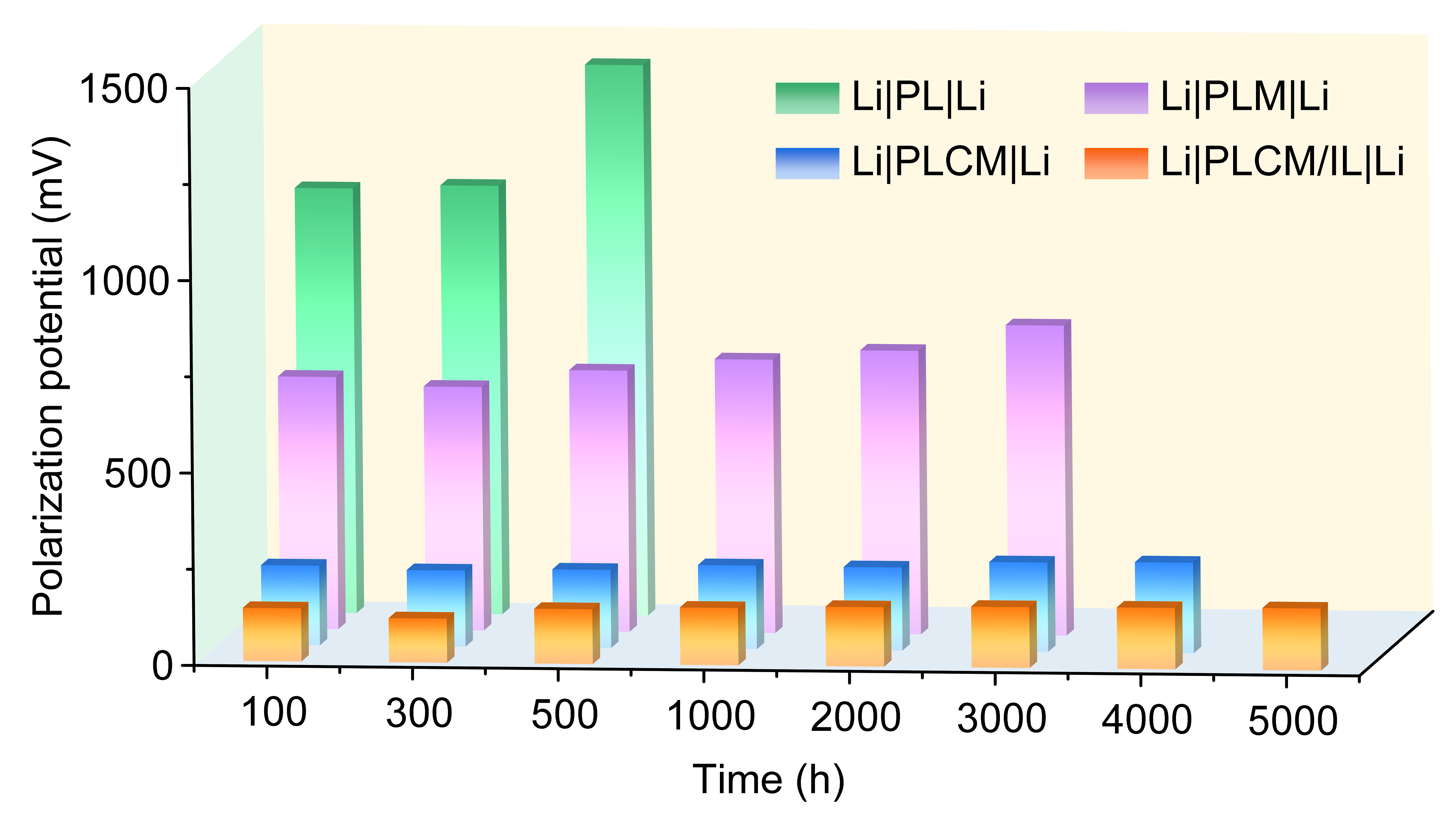


**Figure S12** Polarization potential of Li|CSEs|Li during Li plating/stripping.


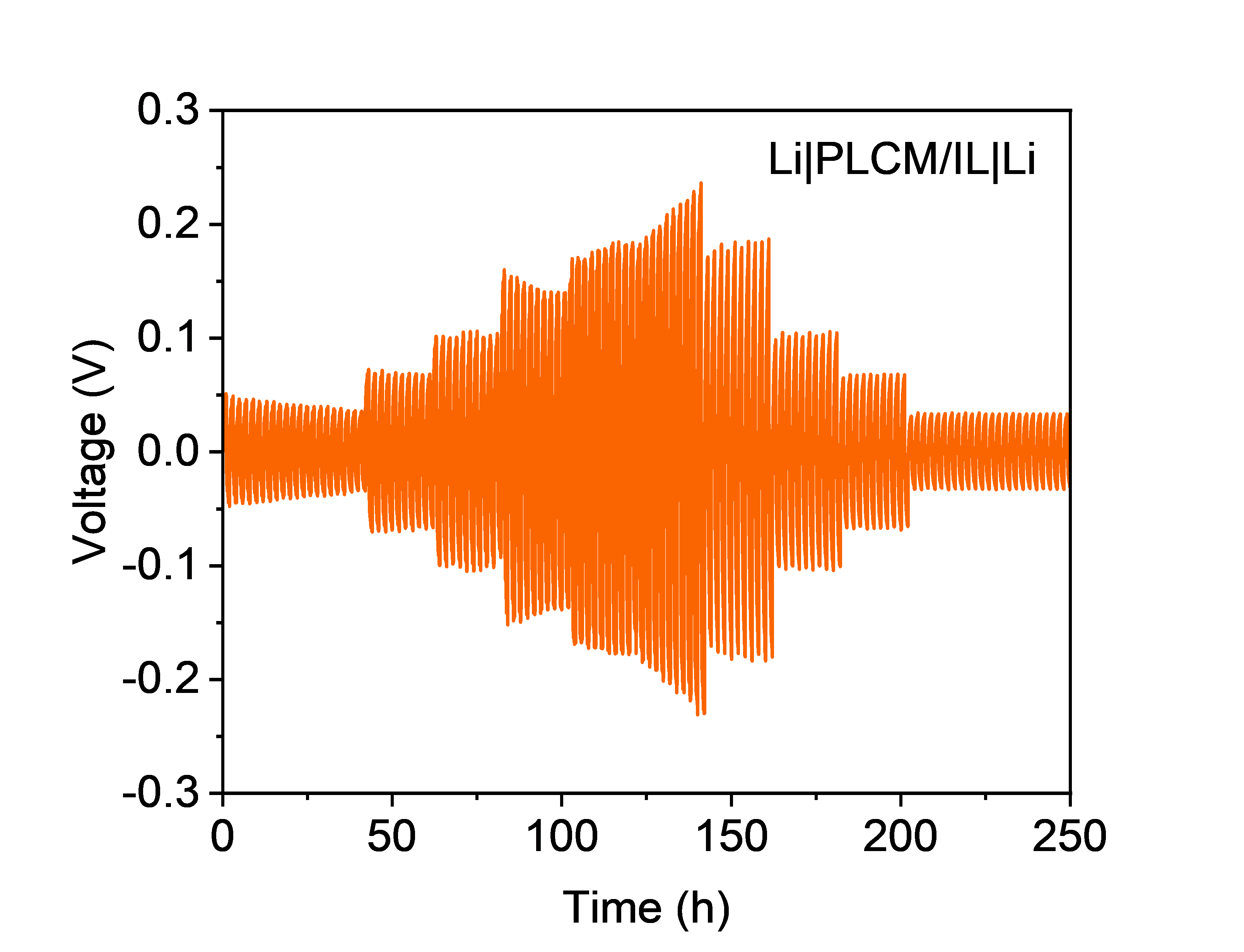


**Figure S13** The rate performance of the Li|PLCM/IL|Li cells under current densities ranging from 0.1 to 1.0 mA cm^-2^, with each cycle maintained for 2 h.


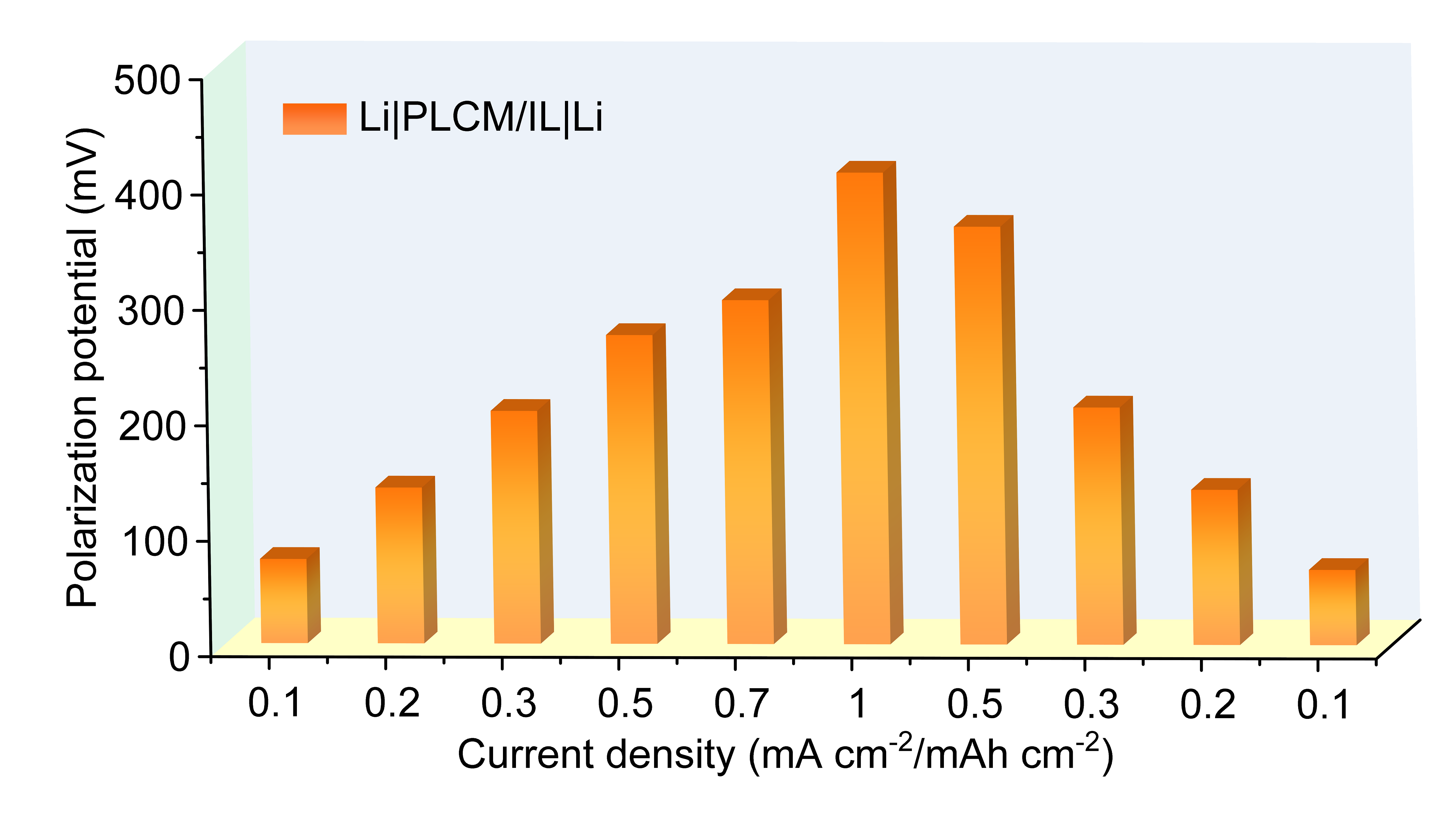


**Figure S14** Polarization potential of Li|PLCM/IL|Li at different current density.


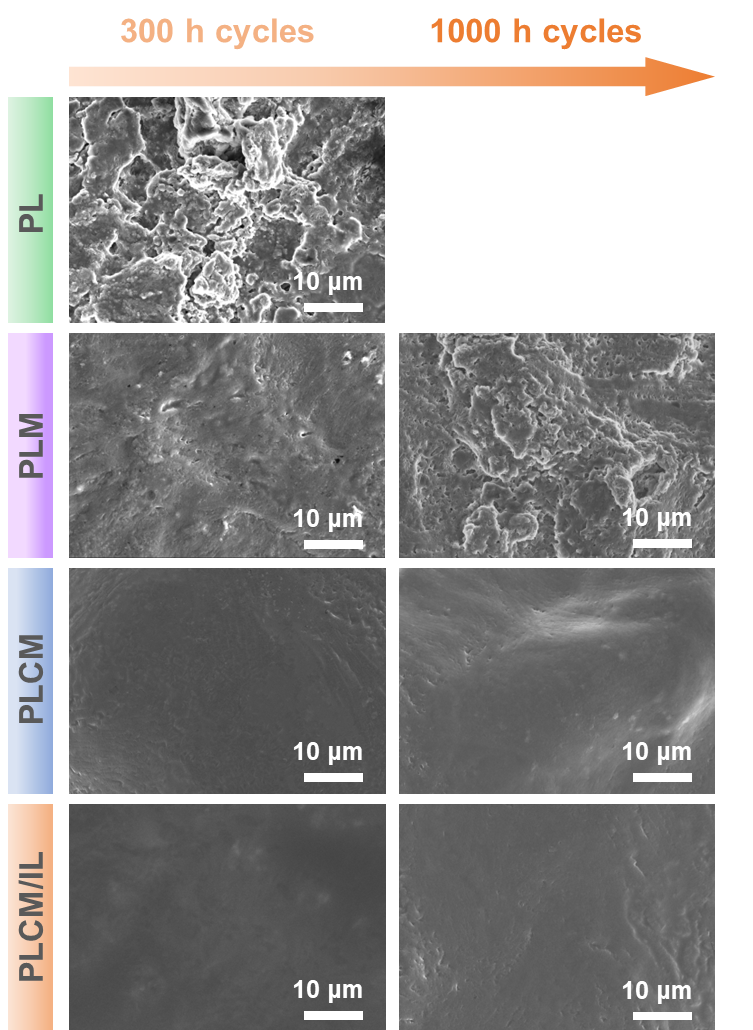


**Figure S15** TOP-view SEM images of Li anodes after 300 h and 1000 h of Li symmetric cells cycling.


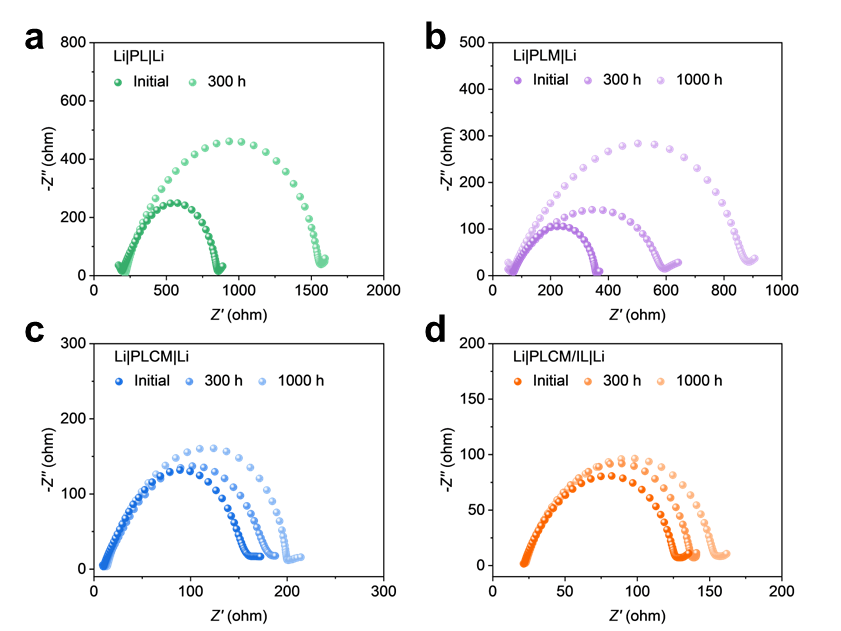


**Figure S16** EIS curves before and after 300 h and 1000 h of Li|CSEs|Li cells cycling.


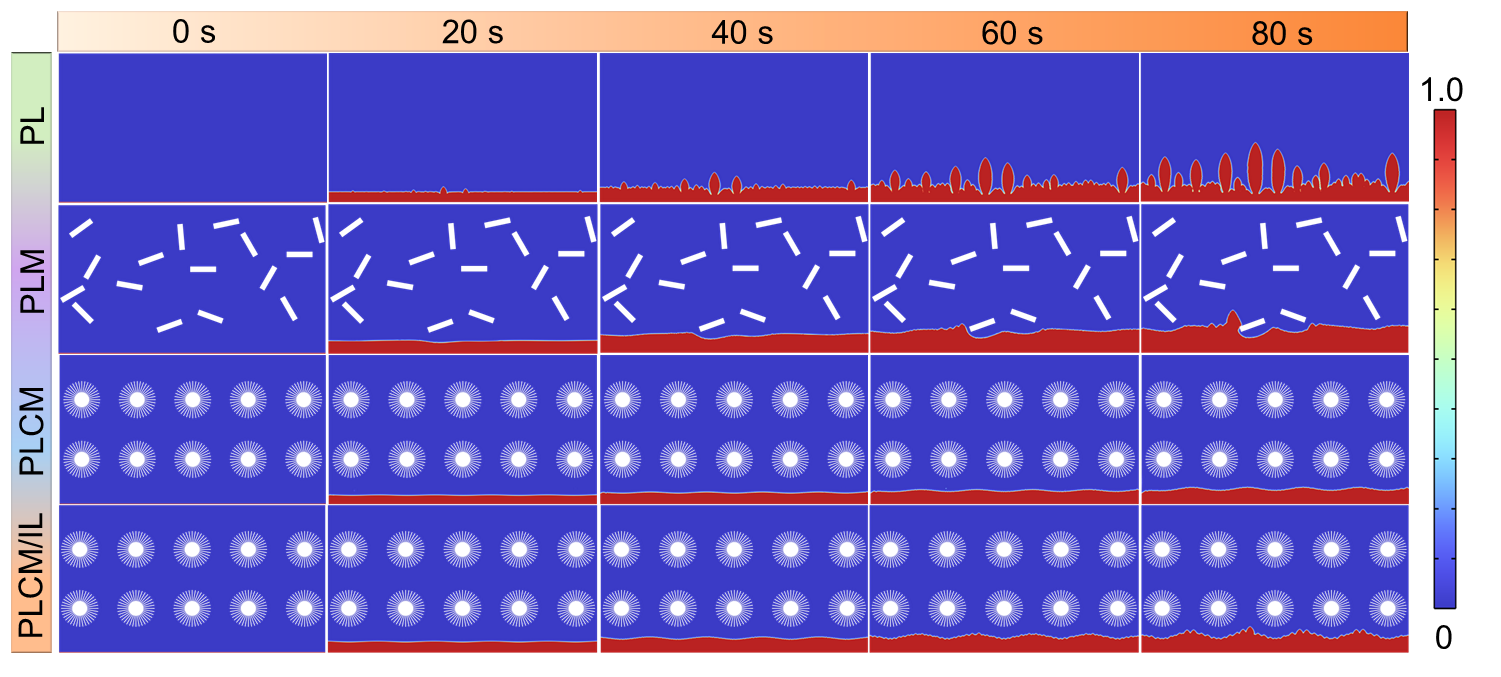


**Figure S17** The snapshots of order parameter at various evolution times.


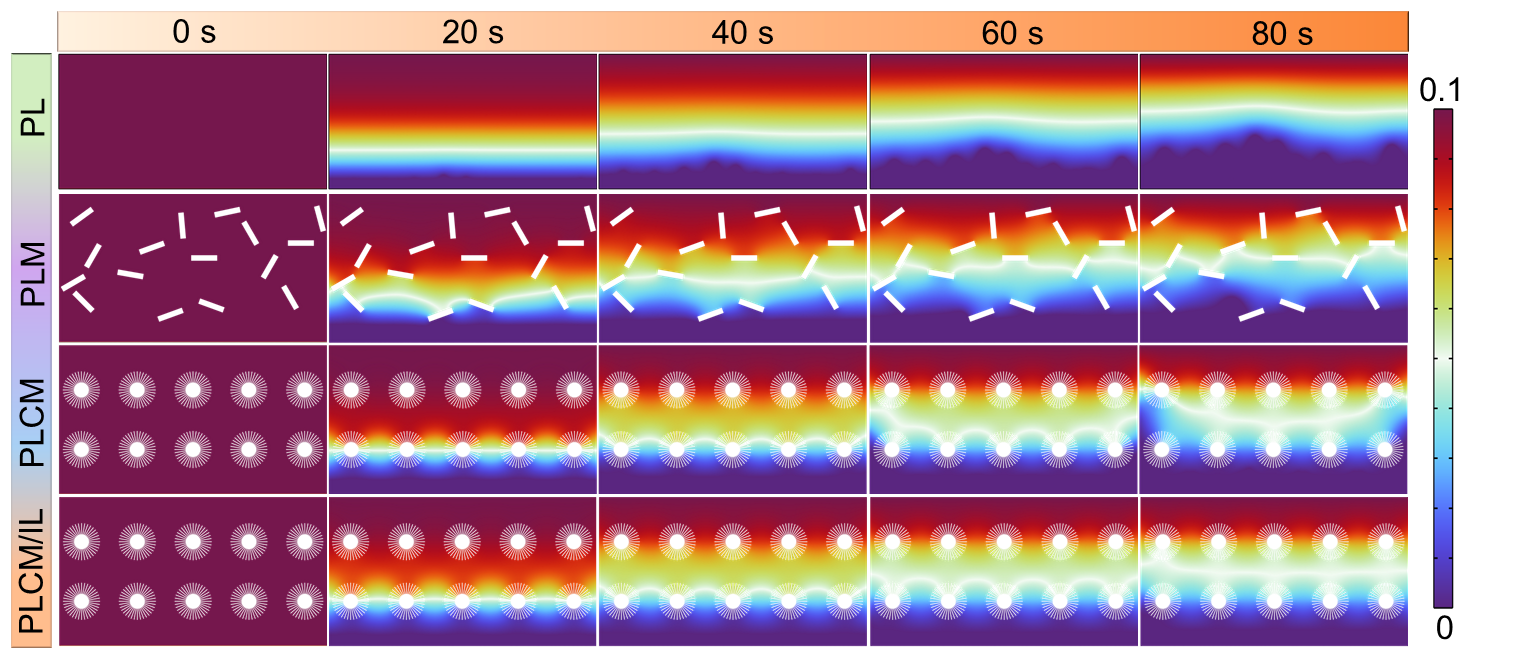


**Figure S18** The snapshots of electric potential at various evolution times.


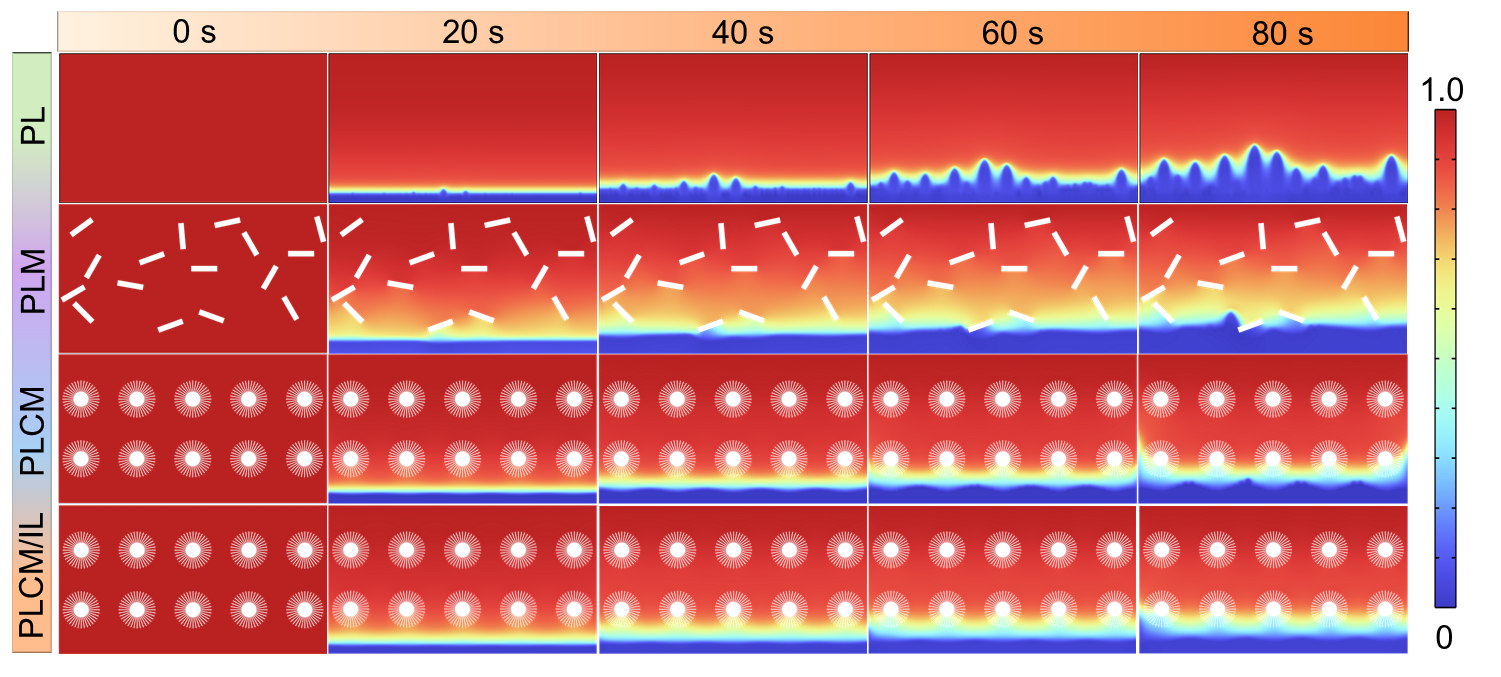


**Figure S19** The snapshots of Li^+^ concentration at various evolution times.


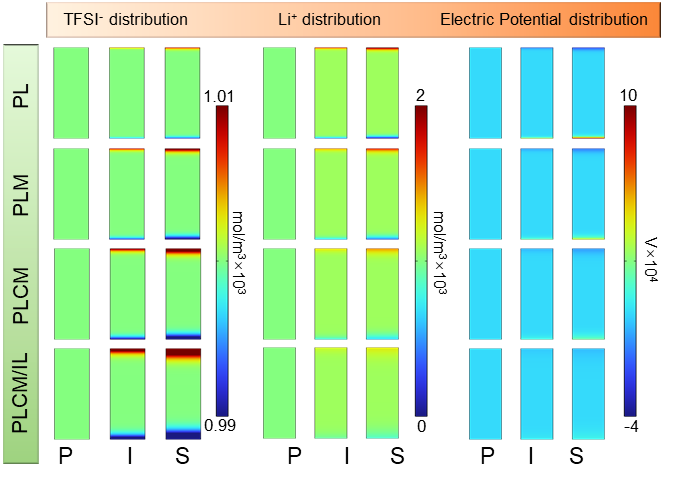


**Figure S20** Distribution of TFSI^-^, Li^+^ and potential in PL, PLM, PLCM and PLCM/IL (P: pristine state, I: intermediate state, S: steady state).


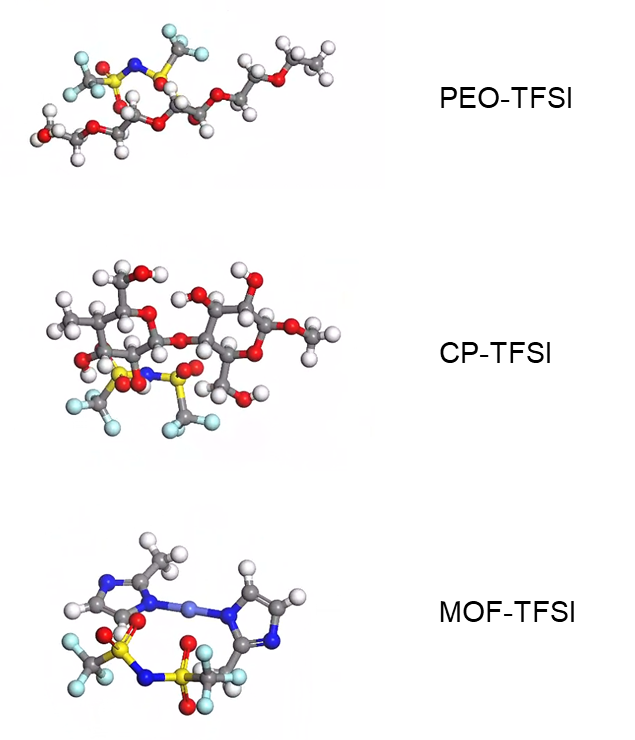


**Figure S21** Atomic structure models of PEO/CP/MOF-TFSI^-^ structural optimization.


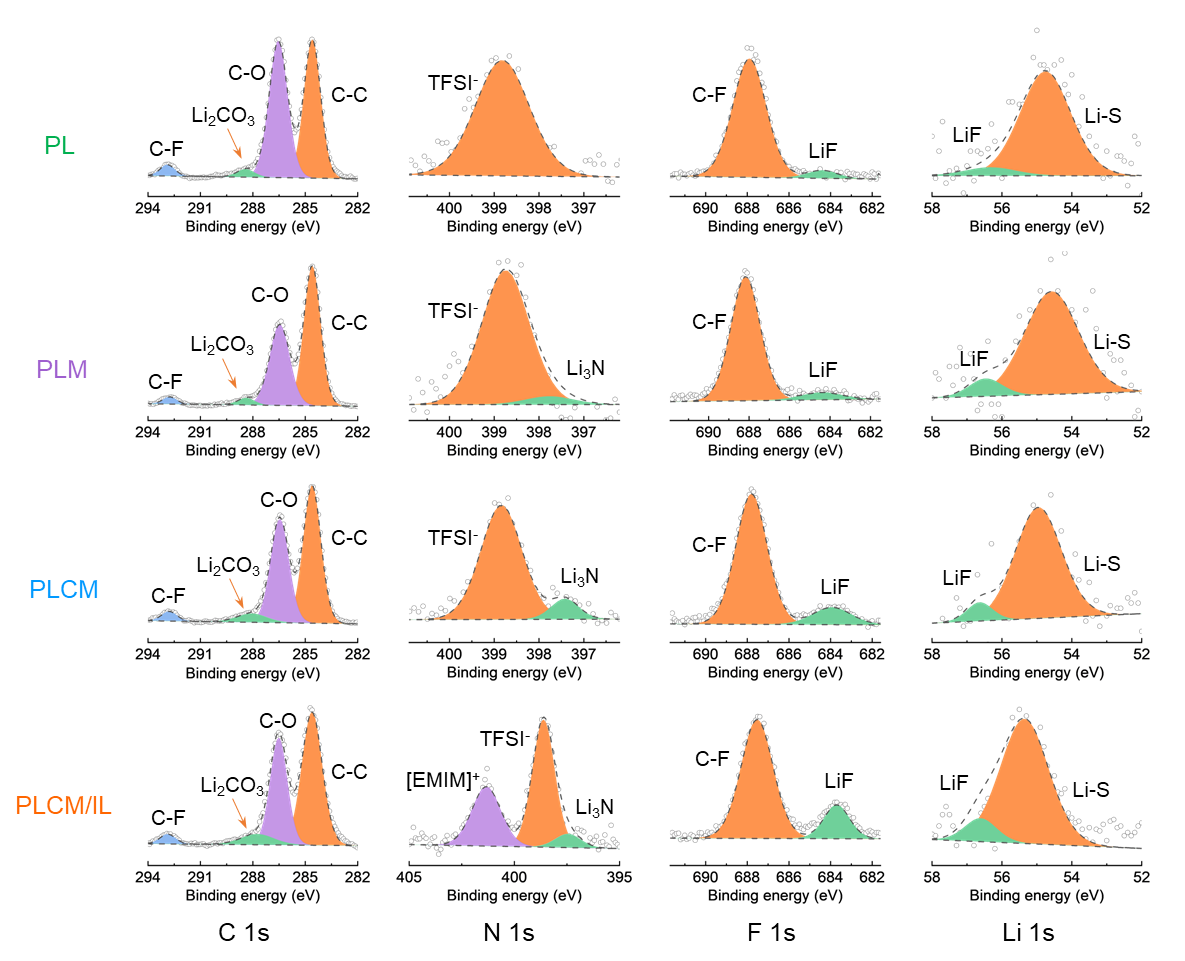


**Figure S22** XPS spectra of C 1s, N 1s, F 1s, and Li 1s of the PL, PLM, PLCM, and PLCM/IL electrolyte surface obtained from Li|Li cells after 100 h of cycling.


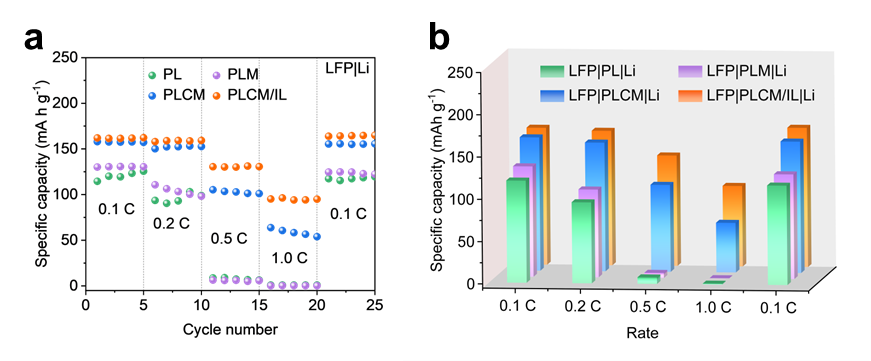


**Figure S23** (a) Rate performance and (b) average specific capacity of LFP|CSEs|Li full cells.

Figure S23 compares the rate performance of different electrolyte cells. The LFP|PLCM/IL|Li cell exhibits high average discharge capacities of 161.8 mAh g^-1^ and 158.9 mAh g^-1^ at 0.1 C and 0.2 C, respectively, and 130.4 mAh g^-1^ at 0.5 C, and 95.1 mAh g^-1^ at 1.0 C. When the rate is reduced back to 0.1 C, the average specific capacity is almost fully recovered (164.5 mAh g^-1^). In contrast, the PL and PLCM cells exhibit significantly lower discharge specific capacities, particularly at 0.5 C and 1.0 C, where they are nearly zero, limiting their commercial viability.


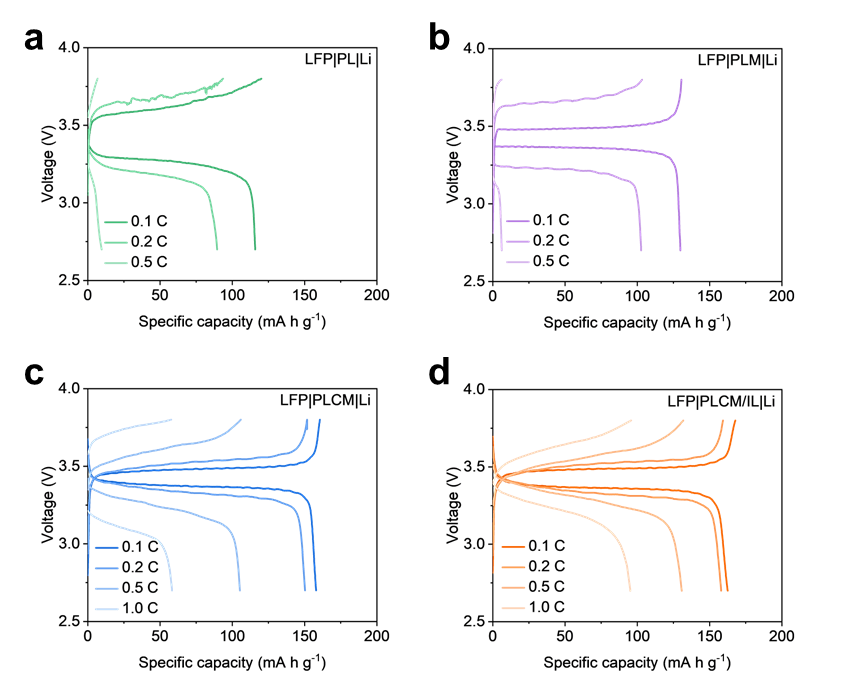


**Figure S24** Charge and discharge voltage-capacity curves at different rates of (a) LFP|PL|Li, (b) LFP|PLM|Li, (c) LFP|PLCM|Li and (d) LFP|PLCM/IL|Li cell.


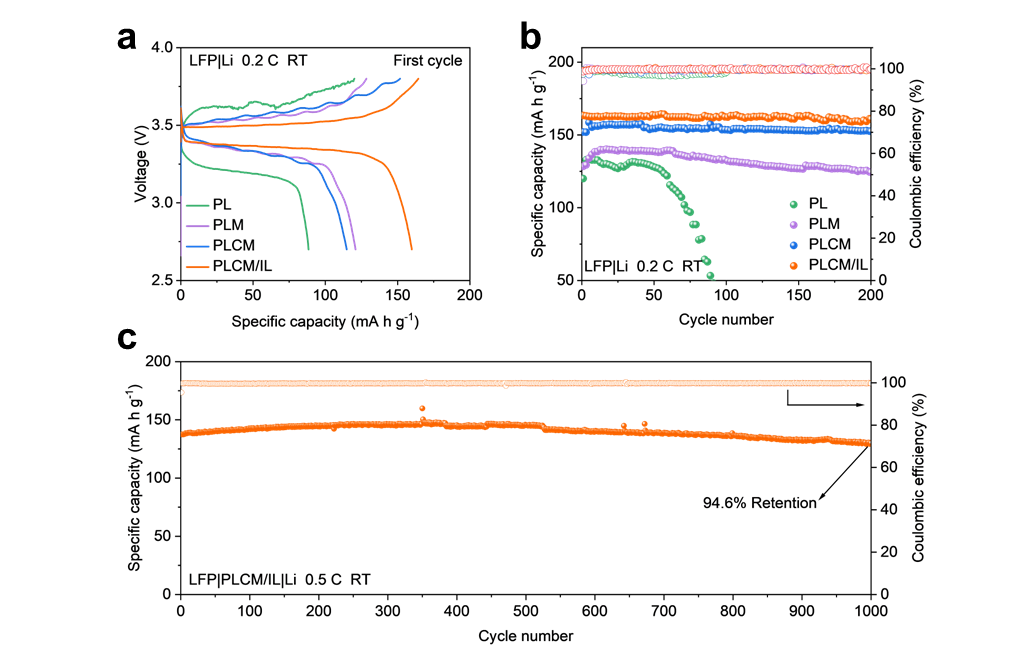


**Figure S25** Electrochemical properties of solid-state LFP|Li cell. (a) Voltage-capacity curves of the LFP|CSEs|Li cells at 0.2 C. Long-term cycling performance of (b) LFP|CSEs|Li full cells at 0.2 C and (c) LFP|PLCM/IL|Li full cell at 0.5 C. The loading of active material on the cathode is ~2.5 mg cm^-2^.


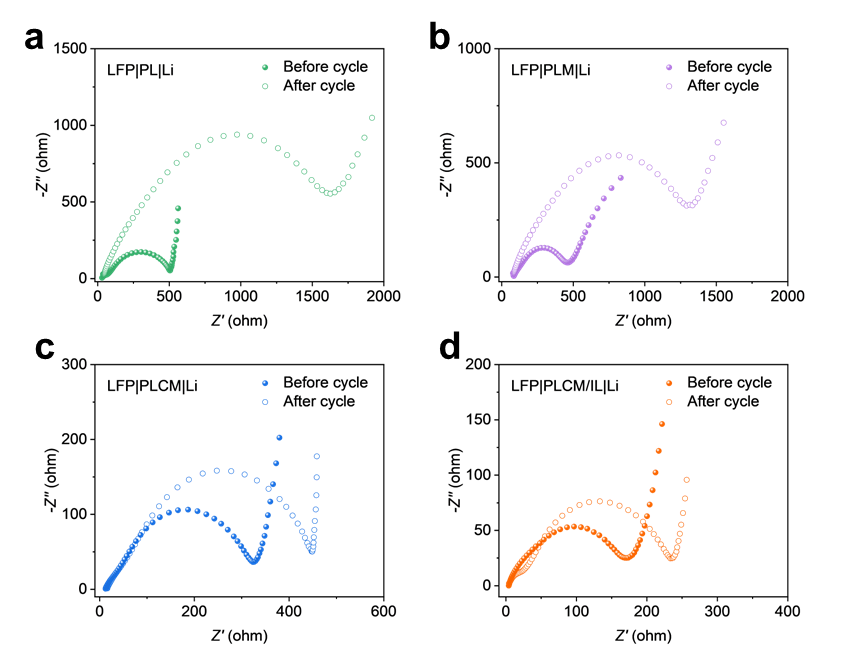


**Figure S26** The EIS curves before and after 50 cycles at 0.2 C with different electrolyte (a) PL, (b) PLM, (c)PLCM and (d) PLCM/IL in LFP|Li cells.


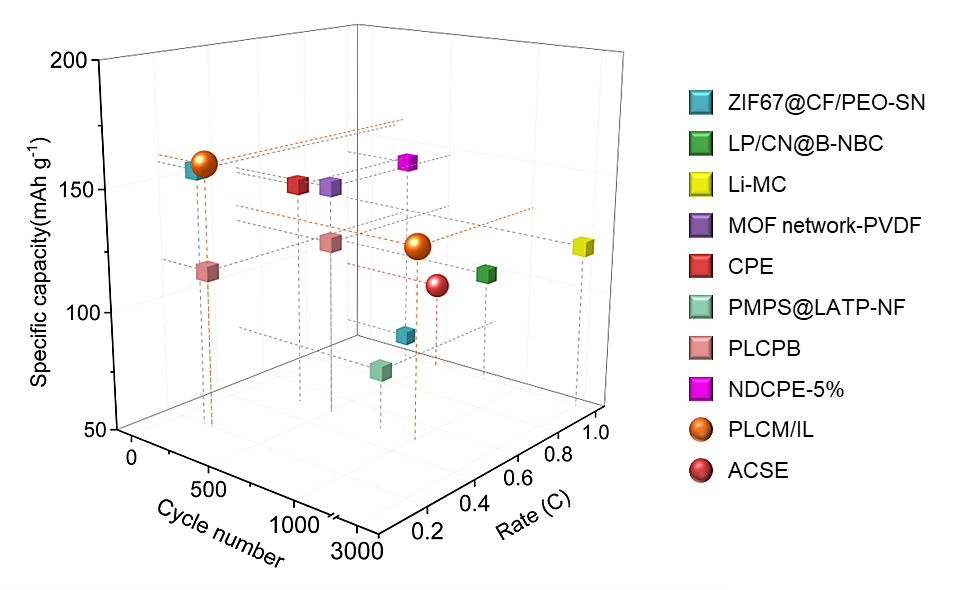


**Figure S27** The comparison of the specific capacity after a certain number of cycles at a fixed rate of the LFP|Li solid-state batteries (see details in Table S3).[1-3,10-14]


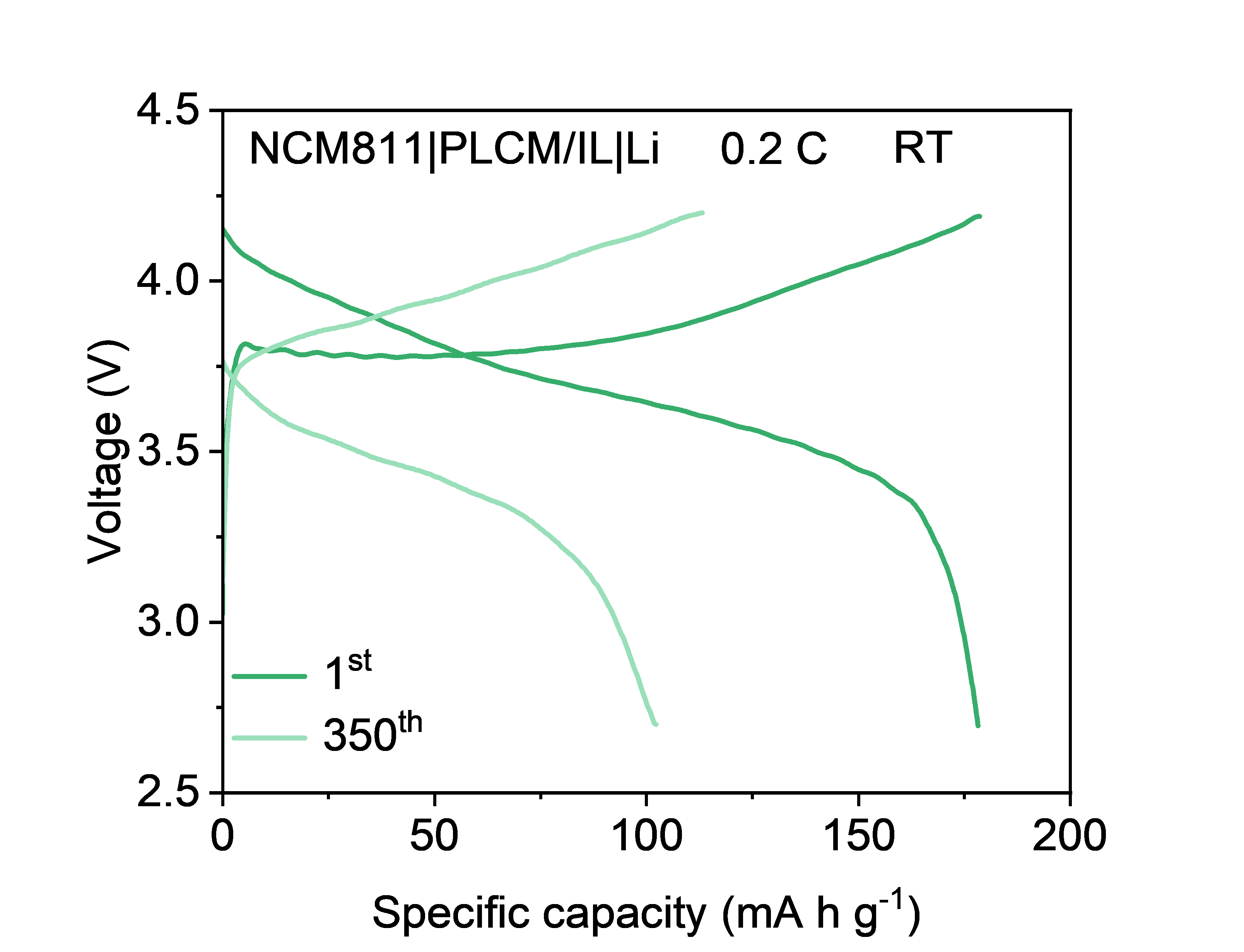


**Figure S28** Voltage-capacity curves of the first and 350^th^ cycle at 0.2 C of NCM811|PLCM/IL|Li cells.


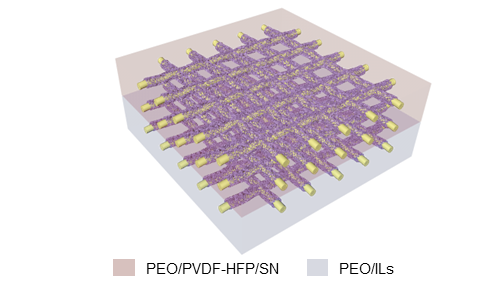


**Figure S29** Schematic diagram of the ACSE electrolyte structure.


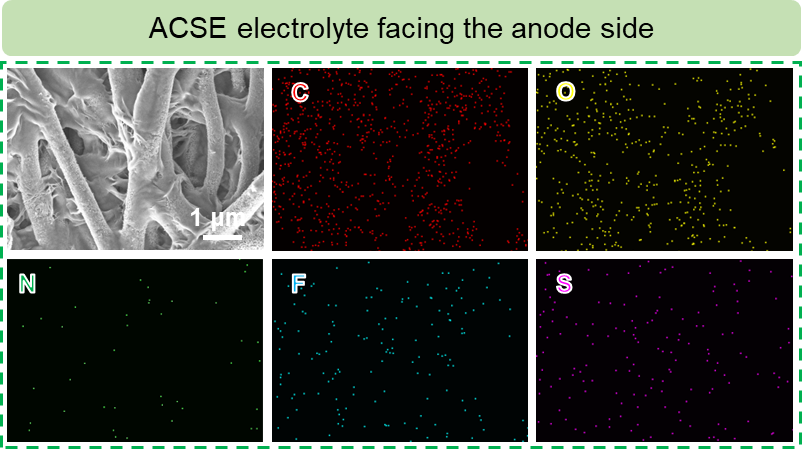


**Figure S30** SEM images and EDS mapping of the ACSE electrolyte facing the anode side.


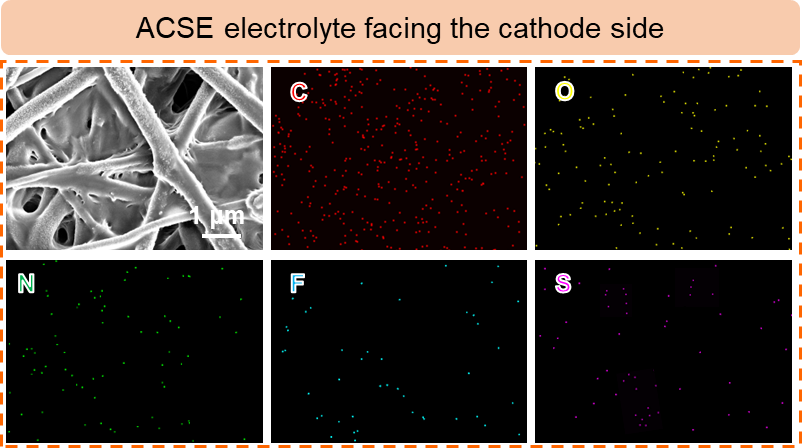


**Figure S31** SEM images and EDS mapping of the ACSE electrolyte facing the cathode side.


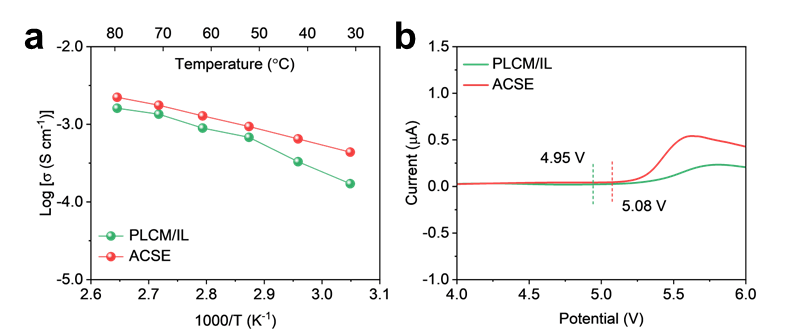


**Figure S32** (a) Ion conductivity and (b) linear sweep voltammetry curve of PLCM/IL and ACSE electrolyte.


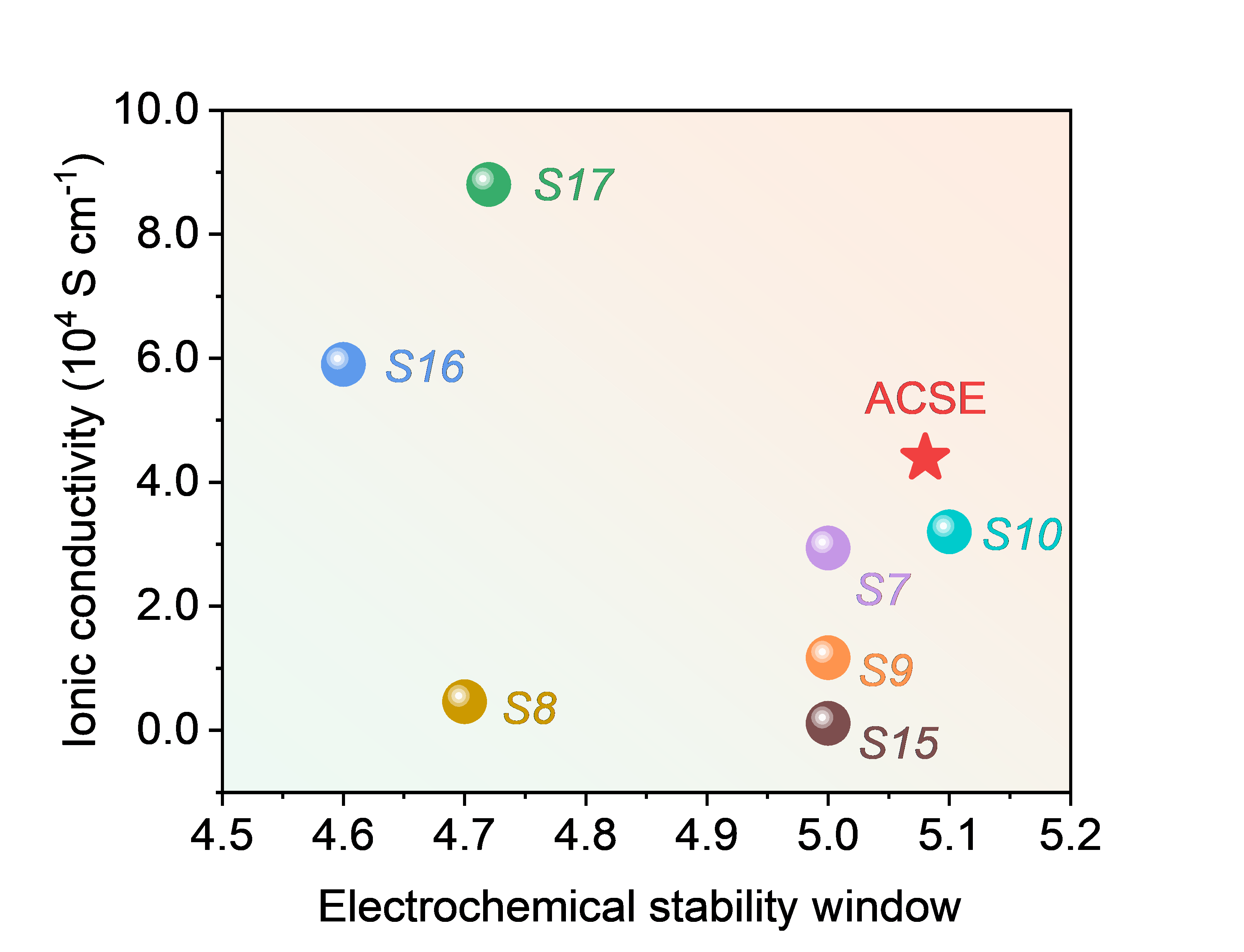


**Figure S33** The comparison of the electrochemical stability window and ionic conductivity of recently reported CSEs[7-10,15-17].


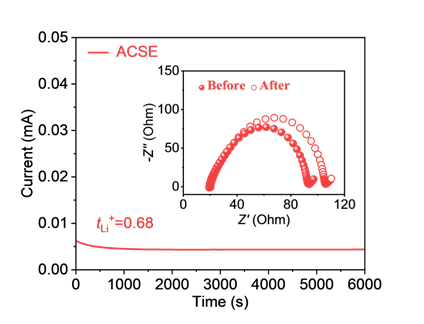


**Figure S34** Li^+^ transference number of ACSE electrolyte.


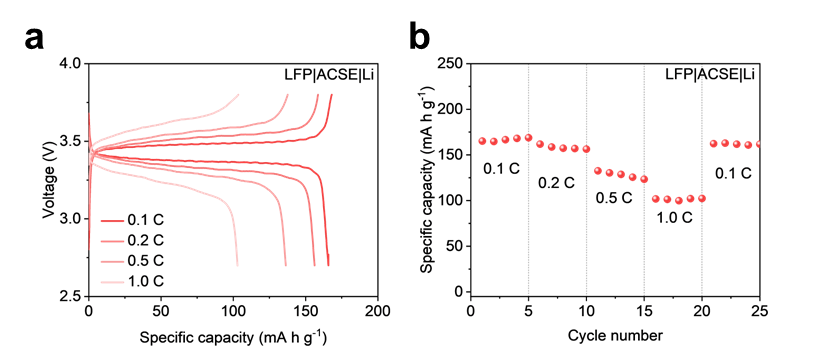


**Figure S35** (a) Voltage-capacity curves under different rates and (b) rate performance of solid-state LFP|ACSE|Li cell. The loading of active material on the cathode is ~2.5 mg cm^-2^.


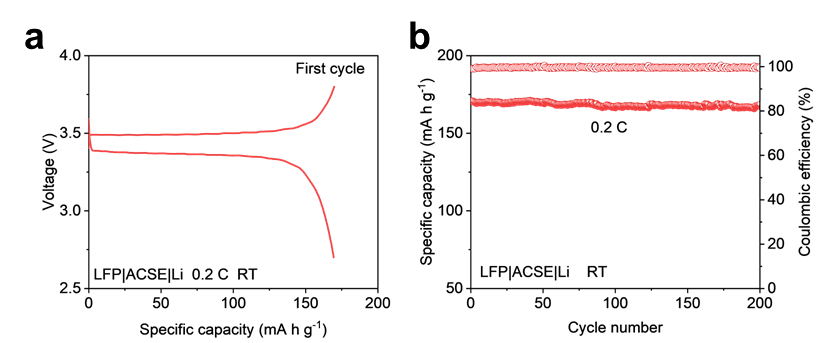


**Figure S36** (a) Voltage-capacity curve of the first cycle and (b) cycling performance at 0.2 C of solid-state LFP|ACSE|Li cell. The loading of active material on the cathode is ~2.5 mg cm^-2^.


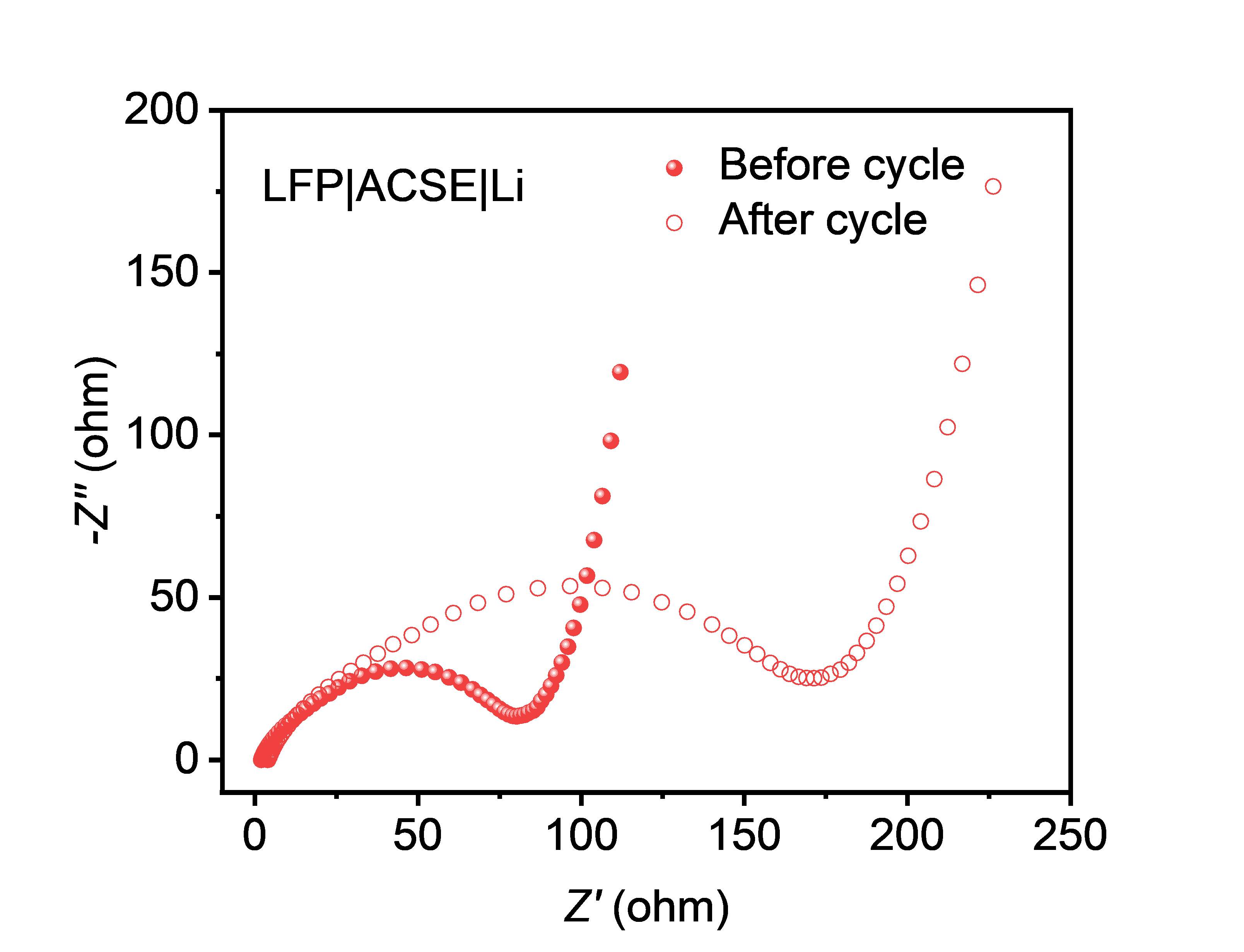


**Figure S37** The EIS curves before and after 50 cycles at 0.2 C with LFP|ACSE|Li cells.


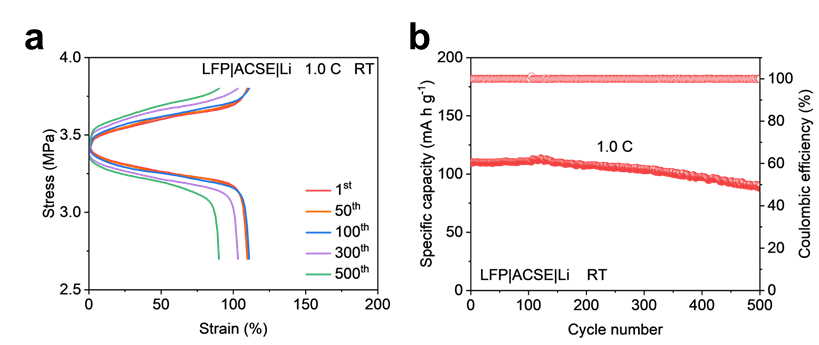


**Figure S38** (a) Charge and discharge voltage curves for the selected cycles (b) cycling performance at the rate of 1.0 C of solid-state LFP|ACSE|Li cell. The loading of active material on the cathode is ~2.5 mg cm^-2^.


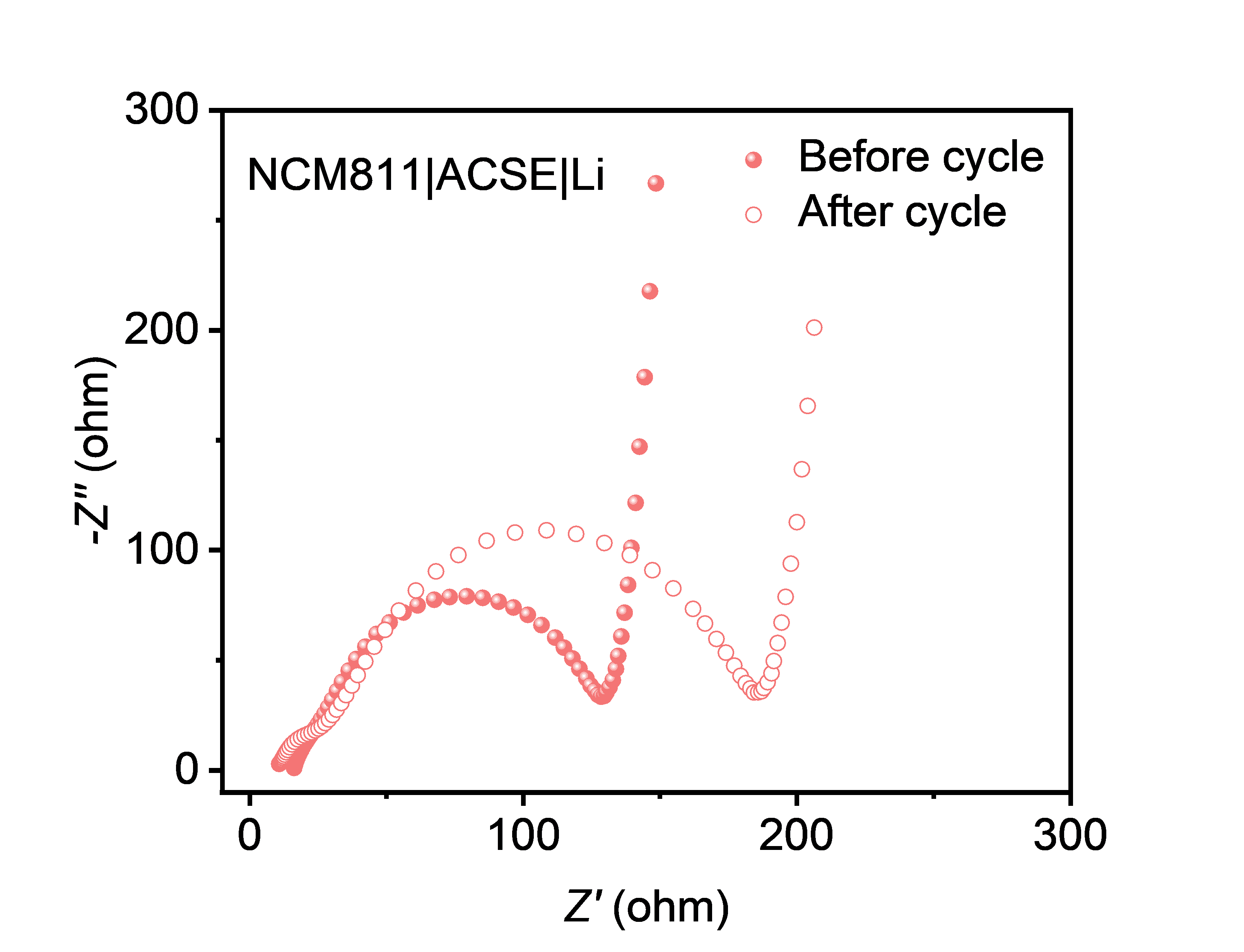


**Figure S39** The EIS curves before and after 50 cycles at 0.2 C with NCM811|ACSE|Li cells.


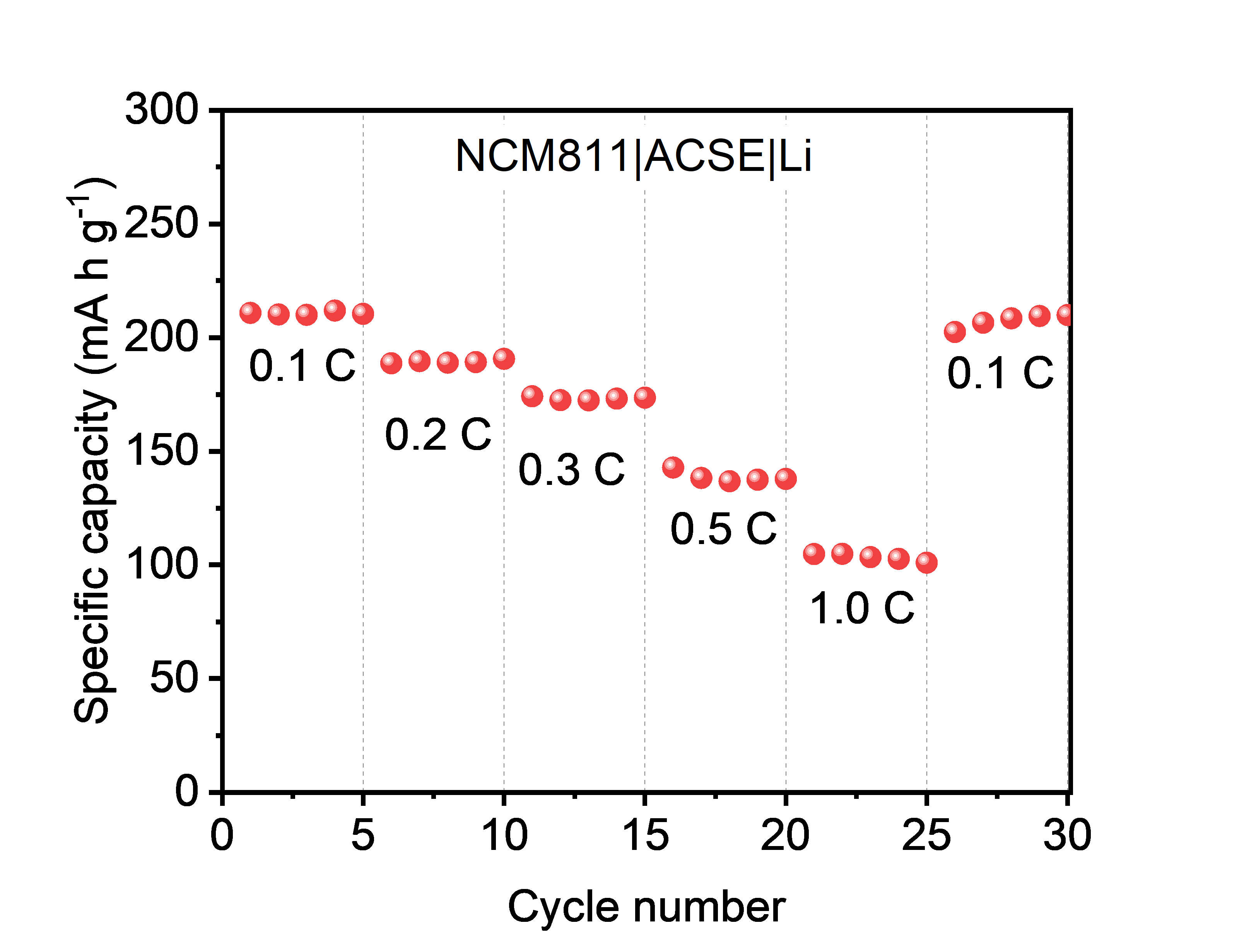


**Figure S40** Rate performance of NCM811|ACSE|Li cell.


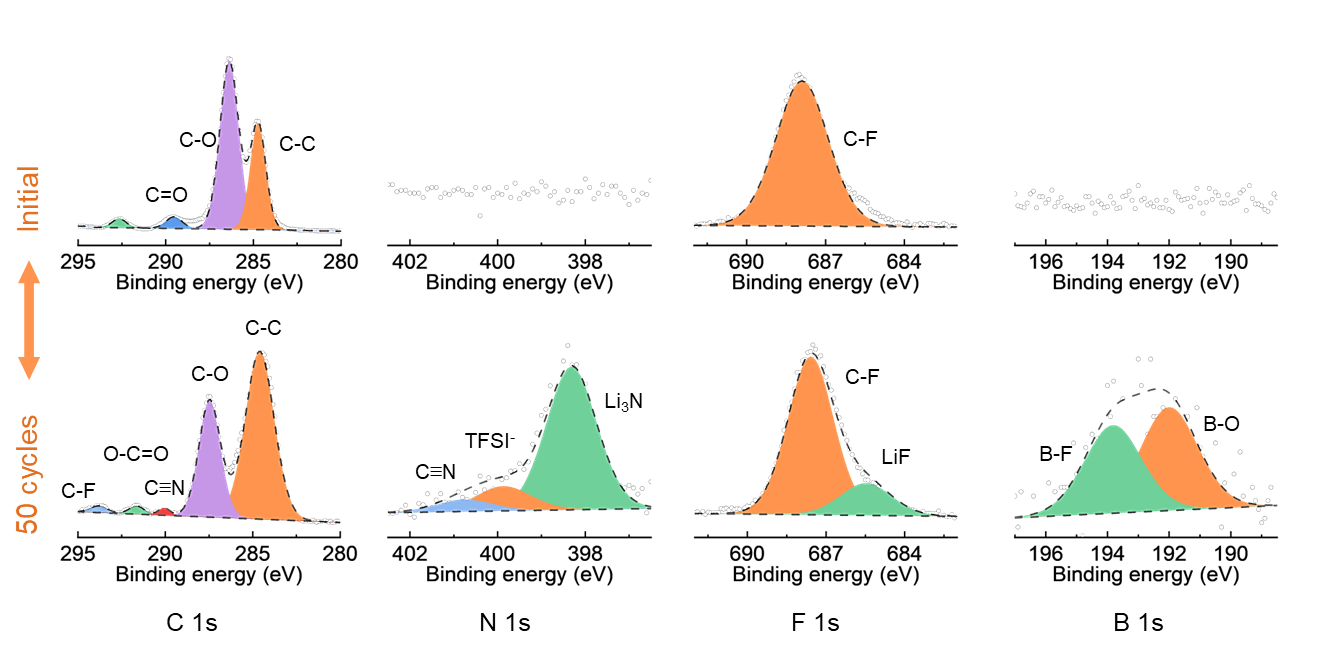


**Figure S41** XPS analysis of NCM811 anode before and after 50 cycles of NCM811/ACSE/Li.


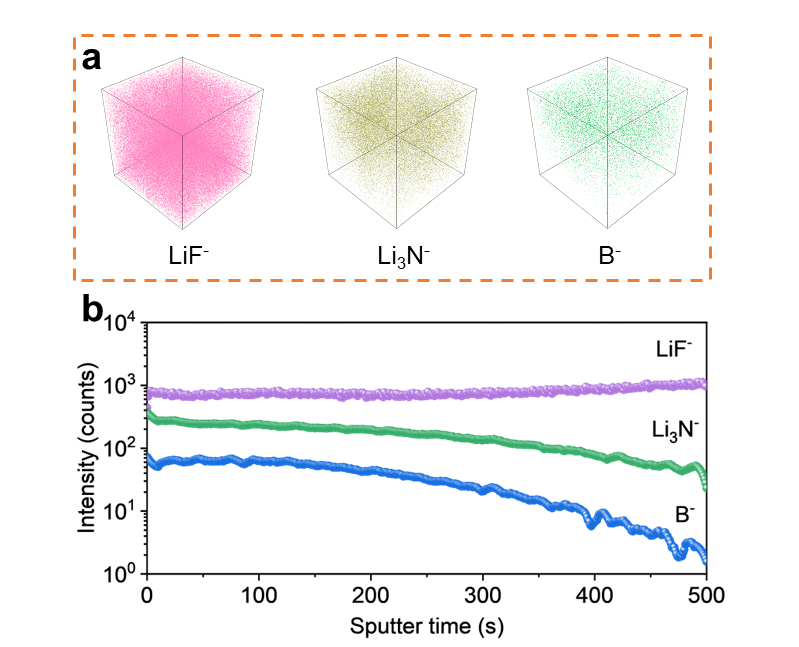


**Figure S42** (a) TOF-SIMS 3D spectra and (b) the corresponding depth profiles of LiF^-^, Li_3_N^-^ and B^-^ on NCM811 anode after 50 cycles of NCM811/ACSE/Li.


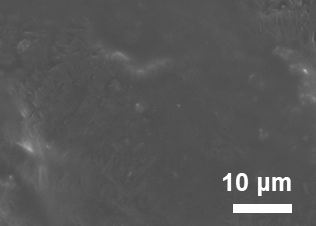


**Figure S43** TOP-view SEM images of Li anode after 50 cycles at 0.2 C (NCM811|ACSE|Li).


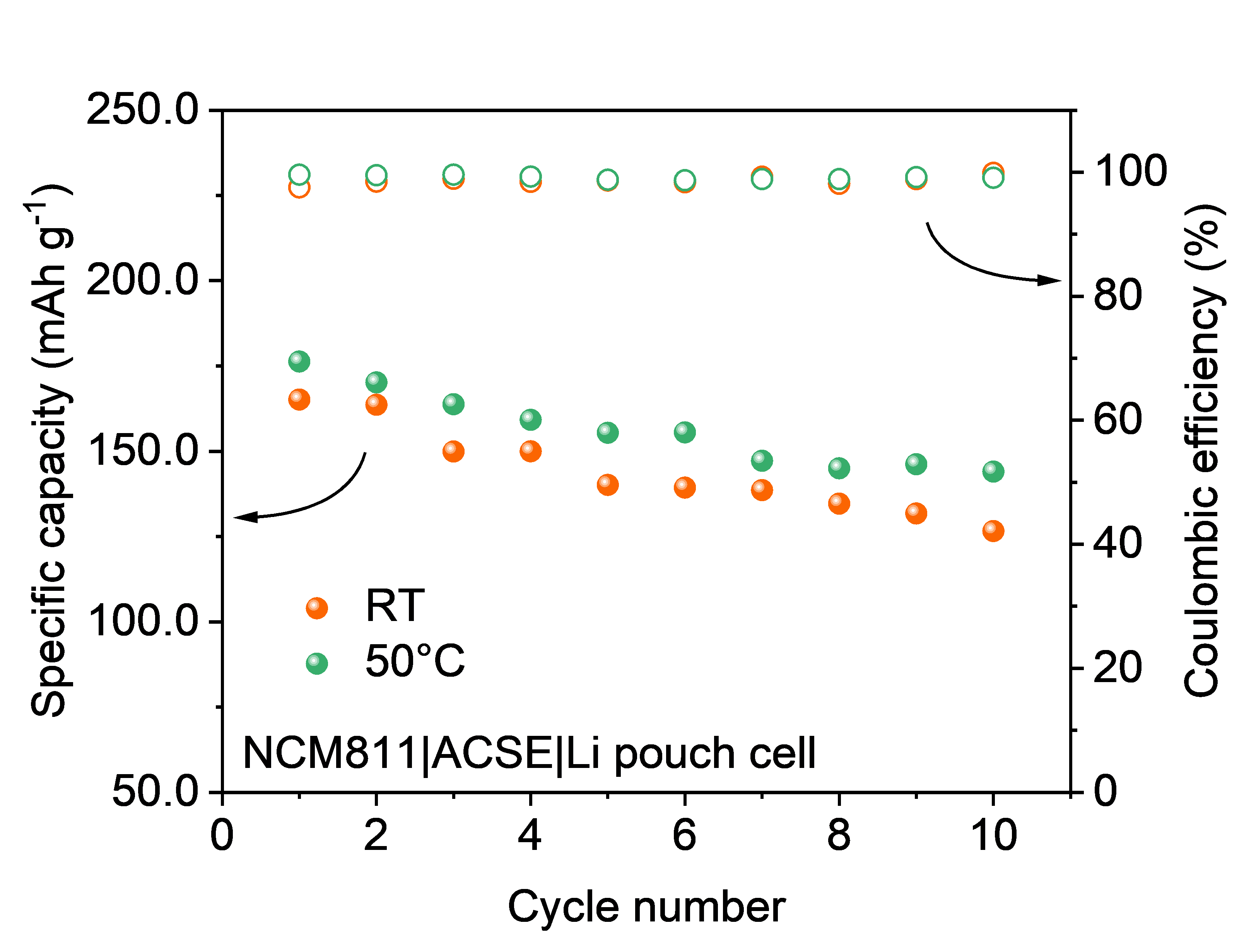


**Figure S44** Cycle performance of the NCM811|ACSE|Li pouch full-cell at room temperature and 50℃.

# 3 Table S1-S4

**Table S1** Glass transition temperature (T_g_), melting temperature (T_m_), and decomposition temperature (T_d_) of PL, PLM, PLCM and PLCM/IL.

| Electrolyte | T_g_ (℃) | T_m_ (℃) | T_d_ (℃) |
| --- | --- | --- | --- |
| **PL** | -39.2 | 56.5 | 191 |
| **PLM** | -38.7 | 52.1 | 257 |
| **PLCM** | -38.4 | 47.6 | 322 |
| **PLCM/IL** | -38.1 | 38.7 | 330 |

**Table S2** The comparison of the Young's modulus of recently reported CSEs with the thickness of the electrolyte.

| Electrolyte | Young's modulus (MPa) | Thickness (μm) |
| --- | --- | --- |
| **ZIF-67@CF/PEO-SN[9]** | 18.66 | 80 |
| **MOF@GF-PVHF[1]** | 1660 | 120 |
| **LP/CN@B-NBC[10]** | 80.4 | 50.5 |
| **GFC@PEO-1wt% MXene[18]** | 315 | 92.9 |
| **LA-PEO-PAM-3-1-1[19]** | 375 | 27 |
| **PLCM/IL** | 1212.3 | 32.5 |

**Table S3** The comparison of the specific capacity after a certain number of cycles at a fixed rate of the LFP|Li solid-state batteries.

| Electrolyte | Cycle number | Rate (C) | Specific capacity (mAh g^-1^) |
| --- | --- | --- | --- |
| **ZIF-67@CF/PEO-SN[1]** | 150  300 | 0.2  1.0 | 156.3  60 |
| **LP/CN@B-NBC[10]** | 800 | 1.0 | 98.7 |
| **Li-MC[3]** | 3000 | 1.0 | 120 |
| **MOF network-PVDF[2]** | 500 | 0.5 | 146.6 |
| **CPE[11]** | 300 | 0.5 | 144.6 |
| **PMPS@LATP-NF[12]** | 800 | 0.5 | 75 |
| **PLCPB[13]** | 200  500 | 0.2  0.5 | 116  123.5 |
| **NDCPE-5%[14]** | 300 | 0.1 | 142 |
| **PLCM/IL** | 200  1000 | 0.2  0.5 | 159.3  130.1 |
| **ACSE** | 500 | 1.0 | 90.4 |

**Table S4** Calculation of the gravimetric/volumetric energy density of NCM811|ACSE|Li pouch cell.

| Component of pouch cell | Parameter | Real value |
| --- | --- | --- |
| **Cayhode (NCM811)** | Active material loading (mg cm^-2^)  NCM811 ratio (%)  Thickness (μm)  Areal capacity (mAh cm^-2^) | 20.3  94.5  90  3.35 |
| **ACSE electrolyte** | Thickness (μm)  Areal density (mg cm^-2^) | 32.5  3.67 |
| **Anode (Li)** | Thickness (μm)  Areal density (mg cm^-2^) | 40  2.14 |
| **Current collector (Al)** | Thickness (μm)  Areal density (mg cm^-2^) | 14  3.78 |
| **Liquid electrolyte** | Areal density (μL cm^-2^)  Areal density (mg cm^-2^) | 5  6.1 |
| **Pouch cell** | Total mass (mg cm^-2^)  Total thickness (μm)  Average discharge voltage (V)  Gravimetric energy density (Wh kg^-1^)  Voltage energy density (Wh L^-1^) | 37.17  176.5  3.75  337.9  711.7 |

# 4 Reference

1. Z. Wang, Y. Hou, S. Li, Z. Xu, X. Zhu, et al. Quasi‐solid composite polymer electrolyte‐based structural batteries with high ionic conductivity and excellent mechanical properties. Small Structures. **5**(8), (2024). <https://doi.org/10.1002/sstr.202400050>

2. L. Du, B. Zhang, W. Deng, Y. Cheng, L. Xu, et al. Hierarchically self‐assembled mof network enables continuous ion transport and high mechanical strength. Advanced Energy Materials. **12**(24), (2022). <https://doi.org/10.1002/aenm.202200501>

3. Y. Zheng, N. Yang, R. Gao, Z. Li, H. Dou, et al. “Tree‐trunk” design for flexible quasi‐solid‐state electrolytes with hierarchical ion‐channels enabling ultralong‐life lithium‐metal batteries. Advanced Materials. **34**(44), (2022). <https://doi.org/10.1002/adma.202203417>

4. P. Pan, M. Zhang, Z. Cheng, L. Jiang, J. Mao, et al. Garnet ceramic fabric-reinforced flexible composite solid electrolyte derived from silk template for safe and long-term stable all-solid-state lithium metal batteries. Energy Storage Materials. **47**(279-287 (2022). <https://doi.org/10.1016/j.ensm.2022.02.018>

5. A. Hu, Z. Sun, Q. Hou, J. Duan, C. Li, et al. Regulating lithium plating/stripping behavior by a composite polymer electrolyte endowed with designated ion channels. Small. **18**(52), (2022). <https://doi.org/10.1002/smll.202205571>

6. G. Wang, P. He, L. Z. Fan. Asymmetric polymer electrolyte constructed by metal–organic framework for solid‐state, dendrite‐free lithium metal battery. Advanced Functional Materials. **31**(3), (2020). <https://doi.org/10.1002/adfm.202007198>

7. C. Li, S. Deng, W. Feng, Y. Cao, J. Bai, et al. A universal room‐temperature 3d printing approach towards porous mof based dendrites inhibition hybrid solid‐state electrolytes. Small. **19**(21), (2023). <https://doi.org/10.1002/smll.202300066>

8. H. Lian, R. Momen, Y. Xiao, B. Song, X. Hu, et al. High ionic conductivity motivated by multiple ion‐transport channels in 2d mof‐based lithium solid state battery. Advanced Functional Materials. **33**(49), (2023). <https://doi.org/10.1002/adfm.202306060>

9. X. Song, K. Ma, J. Wang, H. Wang, H. Xie, et al. Three-dimensional metal–organic framework@cellulose skeleton-reinforced composite polymer electrolyte for all-solid-state lithium metal battery. ACS Nano. **18**(19), 12311-12324 (2024). <https://doi.org/10.1021/acsnano.4c01257>

10. S. Yin, Y. Huang, Y. Liu, L. Cheng, M. Chen, et al. Advanced composite solid electrolyte architecture constructed with amino‐modified cellulose and carbon nitride via biosynthetic avenue. Advanced Functional Materials. **34**(24), (2024). <https://doi.org/10.1002/adfm.202314976>

11. D. Zhang, X. Meng, W. Zhang, J. Mo, Q. Zhao, et al. A thin and ultrahigh‐ionic‐conductivity composite electrolyte with 3d aramid nanofiber networks toward ambient‐temperature lithium metal batteries. Advanced Energy Materials. **15**(9), (2024). <https://doi.org/10.1002/aenm.202403565>

12. X. Yi, Y. Yang, K. Xiao, S. Zhang, B. Wang, et al. Achieving balanced performance and safety for manufacturing all‐solid‐state lithium metal batteries by polymer base adjustment. Advanced Energy Materials. **15**(10), (2025). <https://doi.org/10.1002/aenm.202404973>

13. J. Song, Y. Xu, Y. Zhou, P. Wang, H. Feng, et al. Cellulose-assisted vertically heterostructured peo-based solid electrolytes mitigating li-succinonitrile corrosion for lithium metal batteries. ACS Applied Materials & Interfaces. **15**(17), 20897-20908 (2023). <https://doi.org/10.1021/acsami.2c22562>

14. S. Lv, X. He, Z. Ji, S. Yang, L. Feng, et al. A supertough and highly‐conductive nano‐dipole doped composite polymer electrolyte with hybrid li+‐solvation microenvironment for lithium metal batteries. Advanced Energy Materials. **13**(44), (2023). <https://doi.org/10.1002/aenm.202302711>

15. M. Yao, Q. Ruan, S. Pan, H. Zhang, S. Zhang. An ultrathin asymmetric solid polymer electrolyte with intensified ion transport regulated by biomimetic channels enabling wide‐temperature high‐voltage lithium‐metal battery. Advanced Energy Materials. **13**(12), (2023). <https://doi.org/10.1002/aenm.202203640>

16. Z. Yang, Y. Ye, N. Meng, F. Lian. Adaptive 3d cross‐linked single‐ion conducting polymer electrolytes enable powerful interface for solid state batteries. Angewandte Chemie International Edition. (2025). <https://doi.org/10.1002/anie.202505232>

17. Q. Lv, Y. Jing, B. Wang, B. Wu, S. Wang, et al. Multilayer asymmetric solid polymer electrolyte with modified interface for high-voltage solid-state li metal batteries. Energy Storage Materials. **65**((2024). <https://doi.org/10.1016/j.ensm.2023.103122>

18. Y.-Q. Mao, G.-H. Dong, W.-B. Zhu, Y.-Q. Li, P. Huang, et al. Novel sandwich structured glass fiber cloth/poly(ethylene oxide)-mxene composite electrolyte. Nano Materials Science. **6**(1), 60-67 (2024). <https://doi.org/10.1016/j.nanoms.2023.01.001>

19. X. Wen, Q. Zeng, J. Guan, W. Wen, P. Chen, et al. 3d structural lithium alginate-based gel polymer electrolytes with superior high-rate long cycling performance for high-energy lithium metal batteries. Journal of Materials Chemistry A. **10**(2), 707-718 (2022). <https://doi.org/10.1039/d1ta07252h>
